# Lipocalin2 suppresses metastasis of colorectal cancer by attenuating NF-κB-dependent activation of snail and epithelial mesenchymal transition

**DOI:** 10.1186/s12943-016-0564-9

**Published:** 2016-12-03

**Authors:** Meibao Feng, Jieqiong Feng, Wuzhen Chen, Wubin Wang, Xuesong Wu, Jing Zhang, Fangying Xu, Maode Lai

**Affiliations:** 1 Department of Pathology, School of Medicine, Zhejiang University, 866 Yuhangtang Road, Hangzhou, Zhejiang 310058 China; 2 Key Laboratory of Disease Proteomics of Zhejiang Province, Hangzhou, Zhejiang China

**Keywords:** Colorectal cancer, Epithelial mesenchymal transition, Lipocalin2 -NF-κB- metastasis, Prognosis

## Abstract

**Background:**

Lipocalin2 (LCN2) is a secretory protein that is aberrantly expressed in several types of cancer and has been involved in metastatic progression. However, neither mechanisms nor the role that LCN2 plays in the metastasis of colorectal cancer are clear.

**Methods:**

LCN2 expression in colorectal cancer was detected by immunohistochemistry in 400 tissue specimens and Kaplan-Meier survival analysis was performed. In vitro, real-time PCR, western blot, colony formation assay, immunofluorescence assay, wound healing assay, migration and invasion experiment were performed to investigate the effects of LCN2 in epithelial mesenchymal transition (EMT), migration and invasion, respectively. In vivo mouse xenograft and metastasis models were utilized to determine tumorigenicity and metastasis ability, and immunohistochemistry, real-time PCR, western blot were used to evaluate the related protein expression. Luciferase reporter assay was used to explore the role of LCN2 on NF-ĸB promoter.

**Results:**

LCN2 was highly expressed in 66.5% of the specimens, and significantly correlated with positive E-cadherin in the membrane and negative nuclear β-catenin. Higher expression of LCN2 together with negative NF-κB expression was negatively related to nuclear accumulation of snail and predicted favorable prognosis. LCN2 blocked cell proliferation, migration and invasion in vitro and in vivo, and inhibited translocation of NF-κB into nucleus. NF-κB could reverse the effect of LCN2 on EMT and promote snail expression. Rescued snail expression had similar effect without influencing NF-κB activity.

**Conclusion:**

LCN2 may be an important negative regulator in EMT, invasion and metastasis of CRC via acting as upstream of NF-κB/snail signaling pathway. Thereby combinative manipulation of LCN2 and NF-κB/snail pathway may represent a novel and promising therapeutic approach for the patients with CRC.

**Electronic supplementary material:**

The online version of this article (doi:10.1186/s12943-016-0564-9) contains supplementary material, which is available to authorized users.

## Background

Colorectal cancer (CRC) is the third most common malignancy worldwide and the fourth leading cause of cancer-related deaths globally [1]. Although great advances in chemotherapy and radiotherapy in recent decades have reduced the recurrence and improved survival of CRC, 136 830 new cases were diagnosed and 50 310 deaths attributed to CRC in 2014. Invasion and metastasis are the leading cause for CRC-related mortality. The 5-year survival rate for patients with distant metastasis is only 13% [2].

Lipocalin2 (LCN2, also known as neutrophil gelatinase-associated lipocalin), is dysregulated in a variety of cancers such as pancreatic cancer [3], prostate cancer [4], and hepatocellular carcinoma [5]. It was initially considered as a defender of innate immunity for its capacity to sequester iron, which blocked bacteria growth [6]. Recently, it has been reported that LCN2 is involved in cancer progression. However, whether the role of LCN2 is pro- or anti-metastasis remains controversial, and its underlying mechanisms have not been clarified. For example, LCN2 prevented metastasis and epithelial mesenchymal transition (EMT) in hepatocellular carcinoma [7] and was a novel suppressor of invasion and angiogenesis in pancreatic cancer [8]. Interestingly, LCN2 has also been correlated with disease advancement [9] and appears to modulate several pathways such as the ERK/slug pathway in prostate cancer [10] and the Met/FAK pathway in liver cancer [11] to contribute to EMT progression.

EMT, a vital process, is regarded as a fundamental program in the metastatic cascade by regulating motility and invasion. During this progress, cells lose their epithelial traits, including cell-cell adhesion proteins, like E-cadherin and Zonula Occludens 1 (ZO-1) and acquire mesenchymal traits such as promigratory cytoskeletal proteins like Vimentin and proteases such as matrix metalloproteinase 9 (MMP9) [12, 13]. Cells with the EMT phenotype can even invade as single cells [14, 15]. Thus, exploring the regulatory mechanisms in this progress is essential and crucial to improve patient survival.

Among several critical factors involved in EMT progression, NF-κB was the essential factor that orchestrated the inflammatory process [16], as well as manipulated the initiation and development of a series of cancers. During the process, NF-ĸB could induce several transcriptional factors which potentiated EMT and metastasis of cancers like snail [17], twist [18] and ZEB2 [19]. We hypothesized that LCN2 may modulate EMT and metastasis that were contributed by NF-κB pathway. Notably, the regulation of the NF-κB pathway by LCN2 has not previously been reported.

Here, we provide the first evidence that LCN2 acted upstream of the NF-κB/snail pathway to prevent cancer progression. The results showed that LCN2 significantly inhibited the NF-κB/snail pathway-induced EMT and metastasis both in vitro and in vivo by attenuating its promoter activity.

## Methods

### Cell culture and reagents

The human CRC cell lines of SW480, SW620, HT-29, and RKO were from the American Type Culture Collection, and 293FT cells were from Life Science (Norway); they were respectively cultured in RPMI 1640 (Gibco, Grand Island, USA) and Dulbecco’s Modified Eagle’s Medium (DMEM, Boster Biological Technology, Co. Ltd, Wuhan, China) supplemented with 10% fetal bovine serum (FBS, Haoyang Bioscience, Tianjin, China) and grown at 37 °C under 5% CO_2_. SW620 and RKO cells that stably expressed LCN2 (designated as SW620-LCN2 and RKO-LCN2; vector control-OB), and SW480 and HT29 cells with LCN2 knockdown (designated as SW480-sh-LCN2 and HT29-sh-LCN2; vector control-SHB) were established by lentiviral shuttle vector (Detail of vector was in part of vector construction).

Leptomycin B (LMB, Cat. No.431050) was purchased from Merck Millipore (Darmstadt, Germany), and incubated with SW480 for 20nM (10 h), SW620-LCN2 and RKO-LCN2 for 40nM (6 h). Bay11-7082 (B5556, Cat. No. 196870) and JSH-23 (J4455, Cat. No. 749886) were from Sigma Aldrich and added to the culture medium at 50 μM, for 3 h and 50 μM for 1 h, respectively.

### Vector construction and small interfering RNA knockdown (siRNA)

The lentiviral shuttle vector pLVX-IRES-ZsGreen1-LCN2 was constructed to stably overexpress LCN2. The EGFP-C2 vector containing green fluorescence protein (GFP) was constructed to stably overexpress snail, snail (55KD) was a fusion protein (snail-30KD and GFP). The 597 bp coding sequence of human LCN2 (NM_005564) and the 794 bp coding sequence of human snail (NM_005985) was amplified from the cDNA templates of human SW480 and subcloned into the EcoRI and BamHI sites of the pLVX-IRES-ZsGreen1 vector (Clontech Laboratories) and EGFP-C2 vector. The lentiviral shuttle vector pLVX-shRNA2 (Clontech) was used to express shRNA from the U6 promoter. In addition to expressing shRNA, pLVX-shRNA2 also expresses the green fluorescent protein ZsGreen1. The target sequence was: shLCN2-1, 5′-GAGTTCACGCTGGGCAACATTAAGA-3′.

For small interfering RNA (siRNA) knockdown of p65, cells were transfected with either p65 or negative siRNA (GenePharma, Shanghai, China) using the PowerFect siRNA Transfection Reagent (SignaGen Laboratories, Gaithersburg, MD) following the manufacturer’s instruction. The siRNAs of p65 sequences are listed in Additional file 1.

### Western blot assay

Cell culture medium was concentrated by centrifugation at 4000 rpm with Amicon Ultra-15 (Millipore, Billerica, MA) for 25 min. Cells were scraped, washed 3 times with PBS, and then lysed in buffer consisting of 7 M urea, 2 M thiourea, 4% CHAPS, 65 mM DTT, and 0.2% Bio-lyte (pH3–10, Bio-Rad, Hercules, California, USA) by sonication on ice. The lysates were centrifuged at 12000 rpm for 1 h at 4 °C. Lysate from fresh xenograft tissues was prepared with a DNA/RNA/Protein Isolation kit (R6734-02, Omega) and the nuclear protein was extracted using a Nucleoprotein and Cytoplasm Protein Extract Kit (Kaigen Bioscience, Nanjing, China) following with manufacturer’s instructions.

Subsequently, the protein concentrations were determined by the Bradford method, and aliquots of the protein samples were stored at −80 °C.

Aliquots of protein extracts (15–50 μg) were separated on 10–12% SDS-polyacrylamide gels according to the protein molecular weights. Then, the proteins were electrophoretically transferred onto NC membranes (Bio-Rad, Laboratories, Hercules, California, USA). After blocking with TBS-Tween 20 (TBST) containing 5% low-fat milk, the membranes were incubated with primary antibody overnight at 4 °C. The primary antibodies are listed in Additional file 2. The membranes were then incubated with secondary antibodies (Odyssey, Li-COR, Bioscience, Lincoln, USA) for 1 h at room temperature. Finally, the membranes were developed with the Odyssey system. Loading differences were normalized to a monoclonal GAPDH antibody.

### Reverse transcription-polymerase chain reaction (PCR) and real-time PCR

Total RNA was extracted from cells using TRIzol (Invitrogen, Carlsbad, CA) according to the manufacturer’s protocol, and its concentration was measured using a spectrophotometer (Eppendorf, Hamburg, Germany). Total RNA (4 μg) was reverse-transcribed using the PrimeScript TM RT Reagent Kit (Takara, Bio, Inc, Otsu, Shiga, Japan). Quantitative real-time PCR was performed using the SYBR Premix Ex TaqTM (Takara, Dalian, China) on a LightCycler480 II (Roche, Diagnostics, Switzerland). The relative expression level was calculated using the 2 − ΔΔCt method and normalized by GAPDH expression. The sequences of the specific primers are listed in Additional file 3.

### Enzyme-linked immunosorbent assay

Quantification of serum LCN2 concentration was performed using a commercially available anti-human LCN2 ELISA kit according to the manufacturer’s instructions (DLCN20, R&D system, Minnesota, USA). The absorbance was measured by a microplate reader (Bio-TEK, ELx 800, Saxony, USA) at 450 nm.

### Immunofluorescence staining

The established cell lines (SW480-SHB, SW480-sh-LCN2, SW620-OB and SW620-LCN2) were seeded on slides, after 24 h they were fixed in 4% paraformaldehyde for 15 min at room temperature and washed three times with PBS, then permeabilized with ice-cold 0.5% Triton ×-100 for 20 min at room temperature, followed by three washes with PBS. The slides were blocked in PBS containing 10% bovine serum albumin for 30 min at room temperature, and then were incubated with antibodies of E-cadherin, vimentin and snail (Additional file 2) overnight at 4 °C. After washing three times with PBS, cells were incubated with Alexa Fluor 546 donkey anti-rabbit IgG (Invitrogen, Eugene, OR) for 1 h at room temperature. Finally, cells were counterstained with 50 mg/ml 4′,6-diamidino-2-phenylindole for 20 min before capturing images with a confocal microscope (LSM-510 Meta, Zeiss, Germany). Assays were performed in triplicate.

### Luciferase reporter assay

Luciferase activity was measured as described previously [20]. 450 ng of PGL3.0-NF-ĸB-luc and 50 ng of Renilla luciferase plasmids or 450 ng of PGL3.0-luc and 50 ng of Renilla luciferase plasmids were co-transfected into 293FT-LCN2-overexpression, 293FT-LCN2-knockdown and corresponding control cells. After 48 h of treatment, luciferase activity was assayed using the Dual Luciferase Assay System (Promega, Madison, WI) according to the manufacturer’s protocol on a Luminometer (Berthold Technologies, Bad Wildbad, Germany). Each firefly luciferase value was corrected for its co-transfected Renilla luciferase value.

### Proliferation assay

For the proliferation assays, 5 × 10^3^ cells were seeded into 96-well plate with 100 μl RPMI 1640 supplemented with 10% FBS, using the Cell Counting Kit (CCK8, Boster Biological Technology, Co. Ltd, Wuhan, China) following the manufacturer’s instructions and detected by a microplate reader (Bio-TEK, ELx 800, Saxony, USA).

### Colony-formation on soft agar

A cell suspension (2 × 10^3^ cells) in 2 ml RPMI 1640 supplemented with 10% FBS and 0.3% agar (Affymetrix, Inc, California, USA) were layered onto 6-cm plates (Corning, Inc, Corning, New York, USA) containing 2 ml RPMI 1640 with 10% FBS and 0.6% agar. Plating was carried out in triplicate and repeated at least three times. After 21 days of growth, colonies were photographed.

### Plate colony-formation assay

SW480-SHB, SW480-sh-LCN2, SW620-OB, and SW620-LCN2 cells were separately seeded into six-well plates at 6000 cells/well. The media were changed three times a week, and after 20 days, the cells were fixed in 4% paraformaldehyde for 30 min, stained with 0.5% crystal violet (Beyotime, Biotechnology, Shanghai, China) for 1 h, rinsed three times with PBS to remove excess dye, then photographed and counted.

### In vivo mouse tumorigenicity and metastasis assays

SW620-OB (5 × 10^6^), SW620-LCN2 (5 × 10^6^), SW480-SHB (1 × 10^7^), and SW480-sh-LCN2 cells (1 × 10^7^) in 100 μl PBS were inoculated subcutaneously into the left shoulder of 4 to 5-week-old BALB/c nude mice. Tumor length and width were measured with calipers, and tumor volumes were calculated according to the equation: V (mm^3^) = ½ [width^2^ (mm^2^) × length (mm)]. Growth curves were plotted based on mean tumor volume within each experimental group at the indicated time points. Tumor growth was observed for 27 days (for SW620-OB and SW620-LCN2 cells) and 16 days (for SW480-SHB and SW480-sh-LCN2 cells), respectively.

For metastasis assay, 4.7 × 10^6^ cells for SW620-OB or SW620-LCN2 cells suspended in 100 μl PBS were injected into mice through the tail vein. Mice were assessed for lung metastasis. All the mice were sacrificed at 10 weeks and the number of metastatic lung nodules was counted.

In vivo tumorigenicity or metastasis experiments were performed using five mice per treatment group respectively.

### In Vitro transwell cell migration/invasion assay

Cell migration and invasion assays were performed in 24-well transwell chambers with 8 μm-pore polycarbonate filter inserts (Corning, Inc, Corning, New York, USA). SW480, SW620 were seeded at a density of 2.5 × 10^5^ each well, and HT29, RKO were seeded at a density of 1.5 × 10^5^ each well in uncoated or Matrigel-coated (BD Biosciences, Franklin Lakes, New Jersey, USA) inserts in 200 μl of serum-free RPMI 1640, respectively. The lower chambers were filled with 600 μl of 10% FBS-supplemented RPMI 1640 as chemoattractant. After 48 h and 72 h, cells on the upper side of the filter were removed and those on the lower surface of the insert were fixed in 4% paraformaldehyde (Sinopharm chemical reagent, Co. Ltd, China) for 15 min and then stained with crystal violet (Beyotime, Biotechnology, Shanghai, China) for 10 min. Migrated cells were then digested in 33% acetic acid and quantified by measuring absorbance at 570 nm with a 96-well plate on a microplate reader (Bio-TEK, ELx 800, Saxony, USA). Three independent experiments were performed.

### Wound healing assay

5 × 10^5^ cells in serum-free RPMI 1640 on six-well plates were rinsed three times with phosphate-buffered saline (PBS) and then a sterile 200 μl pipette tip was used to create three separate, parallel wounds. The wells were then washed several times with PBS to remove floating cells. Representative images of cells migrating into the wounds were captured under a microscope immediately and at 48 h after scratch. Three independent experiments were performed.

### Immunohistochemical staining and scoring

Immunohistochemical staining by Envision method was performed on formalin-fixed paraffin-embedded slides, which had been dewaxed and rehydrated before antigen retrieval step [20]. The antibodies and dilutions are listed in Additional file 2. Each specimen was scored according to the proportion of positive tumor cells: 0, negative or <5%; 1, 5–25%; 2, 26–50%; 3, 51–75%; 4, >75%. Score of NF-κB(p65 in nucleus, *n* = 126), E-cadherin (cytomembrane, *n* = 108), β-catenin (nucleus, *n* = 122), Ki67 (nucleus, *n* = 126) and snail (nucleus, *n* = 124) immunohistochemical staining were from the CRC database in our laboratory. Subsequently, NF-κB, E-cadherin, β-catenin, Ki67 and snail were defined as negative (score 0) and positive (scores 1–4). LCN2 was defined as low (score ≤2) and high (score: 3–4) in survival analysis and comparison of E-cadherin, but was defined as low (score ≤3) and high (score = 4) when compared with β-catenin and Ki67.

### Clinical specimens

All 400 patients (219 males, 181 females) were enrolled with informed consent in accordance with a protocol approved by the Ethics Committee of Zhejiang University. The age at diagnosis ranged from 24 to 91 years (median, 61). There were 207 colon specimens and 193 rectum specimens. Of all cases, 307 were tubular adenocarcinoma and 93 were other types of adenocarcinoma. According to TNM staging system, 79 cases were stage I, 120 were stage II, 160 were stage III, and 41 were stage IV. Follow-up data were obtained from the Xiaoshan tumor registry system database. The follow-up period ranged from 1 to 200 months (median, 52).

### Statistical analysis

The statistical software SPSS (version 19.0; IBM, New York, NY) was applied. For results from cell lines, all data are reported as mean ± S.D. Unpaired Student’s *t* tests were used for normally distributed data and non-parametric Mann-Whitney U-tests were used for non-normally distributed data to compare central tendencies. For results in CRC tissues, comparisons of clinicopathological parameters and EMT markers in the LCN2-low and LCN2-high groups were done by the *χ* ^2^ test or Fisher’s exact test. Correlation coefficients were calculated by the Spearman rank correlation test. The 5-year survival rate was calculated using the Life-table method, and survival curves were drawn using the Kaplan-Meier method and compared with the log-rank test. Significance was set at *P* < .05.

## Results

### Aberrant expression of LCN2 in CRC tissues and cell lines

We previously reported that the LCN2 level was markedly higher (fold change > 20, *P* < .01) in the primary colon cell line SW480 than in the paired metastatic colon cell line SW620 by analyzing the differential secretome based on the LC-MS/MS-based label-free quantitative proteomics approach. The results were verified by western blot in supernatants and lysates of SW480 and SW620 cells [21].

**Fig. 1:**
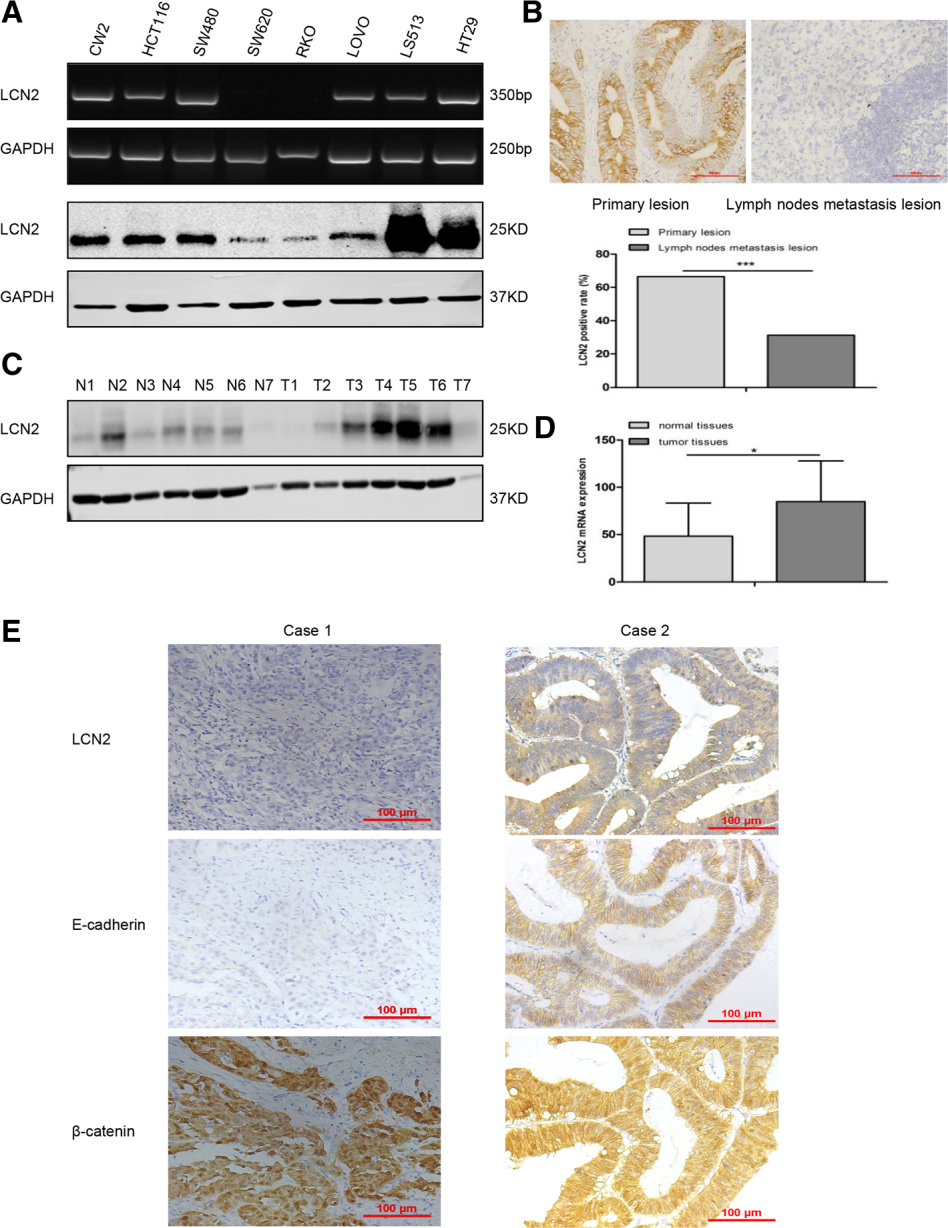
LCN2 expression in CRC cells and tissues. **a** Reverse transcription-polymerase chain reaction (RT-PCR) and western blots of LCN2 expression in the indicated CRC cell lines. **b** LCN2 expression (*upper*), and quantification of LCN2 positive rates (*lower*) in CRC primary lesions and metastatic lymph node lesions (*** *P* < .001). **c** Western blots of LCN2 expression in CRC tissues compared with matched corresponding normal tissues (T, tumor; N, normal). **d** Quantification of LCN2 mRNA expression of normal and tumor tissues in TCGA database (* *P* < .05). (E) Expression of EMT markers (E-cadherin (membrane; *n* = 108, *P* = .036; *r* = 0.213) and β-catenin (nuclear; *n* = 122, *P* = .017; *r* = −0.215) and LCN2 detected by immunohistochemistry in 2 cases of CRC. In case 1, the staining of LCN2 and E-cadherin was weak, as well as membranous β-catenin, but the staining of neuclear β-catenin was strong. Contrary to case 1, the expression of LCN2, E-cadherin, and membranous β-catenin was strong but nuclear β-catenin was weak in case 2. (scale bars, 100 μm)

Furthermore, measurement of LCN2 in a series of CRC lines revealed that LCN2 was highly expressed in CW2, HCT116, SW480, LOVO, LS513 and HT29 cells, but barely expressed in SW620 and RKO cells (Fig. 1a).

Indeed, the detection of LCN2 expression in clinical samples of CRC by immunohistochemistry indicated that the positive rate of LCN2 in primary tumors was significantly higher (66.5%, 266/400) than in lymph node metastases (31.2%, 42/135) (Fig. 1b). Western blot and TCGA database analysis also showed that the expression of LCN2 was higher in tumor tissues than in normal epithelial tissues (Fig. 1c, d). Furthermore, we found higher levels of LCN2 in the serum of 99 CRC patients than in that of the 88 normal controls (Table 1). These results suggested that LCN2 maybe involved in the progression of CRC.

**Table 1:** LCN2 serum levels of CRC patients and normal controls

Serum	Total (n)	Range (ng/ml)	Median (ng/ml)	Average ± SD (ng/ml)	*P* value
Patient	99	32.63–220.9	149.9	165.32 ± 80.95	<0.0001
Normal	88	13.3–129.3	57.8	60.03 ± 25.96	

Interestingly, immunohistochemical analysis of CRC tissues showed that LCN2 was positively correlated with membrane E-cadherin and negatively associated with nuclear β-catenin, which are both critical factors in tumor initiation and progression (Fig. 1e, d and Table 2). This implied that LCN2 expression was positively correlated with the epithelial phenotype in CRC.

**Table 2:** Correlation between the expression of immunohistochemical staining of LCN2 and EMT markers in CRC

Variable	Total (n)	LCN2		*P* value	*r* value
		Lower (n (%))	Higher (n (%))		
E-cadherin (membrane)					
Negative	50	49(45.4)	1(0.9)	0.036	0.213
Positive	58	8(7.4)	50(46.3)		
β-catenin (nuclear)					
Negative	65	47(38.5)	18(14.8)	0.017	−0.215
Positive	57	51(41.8)	6(4.9)		
Ki67					
Negative	21	10(8)	11(8.7)	0.0001	−0.38
Positive	105	92(73)	13(10.3)		

### LCN2 inhibits the proliferation and tumorigenicity of CRC

**Fig. 2:**
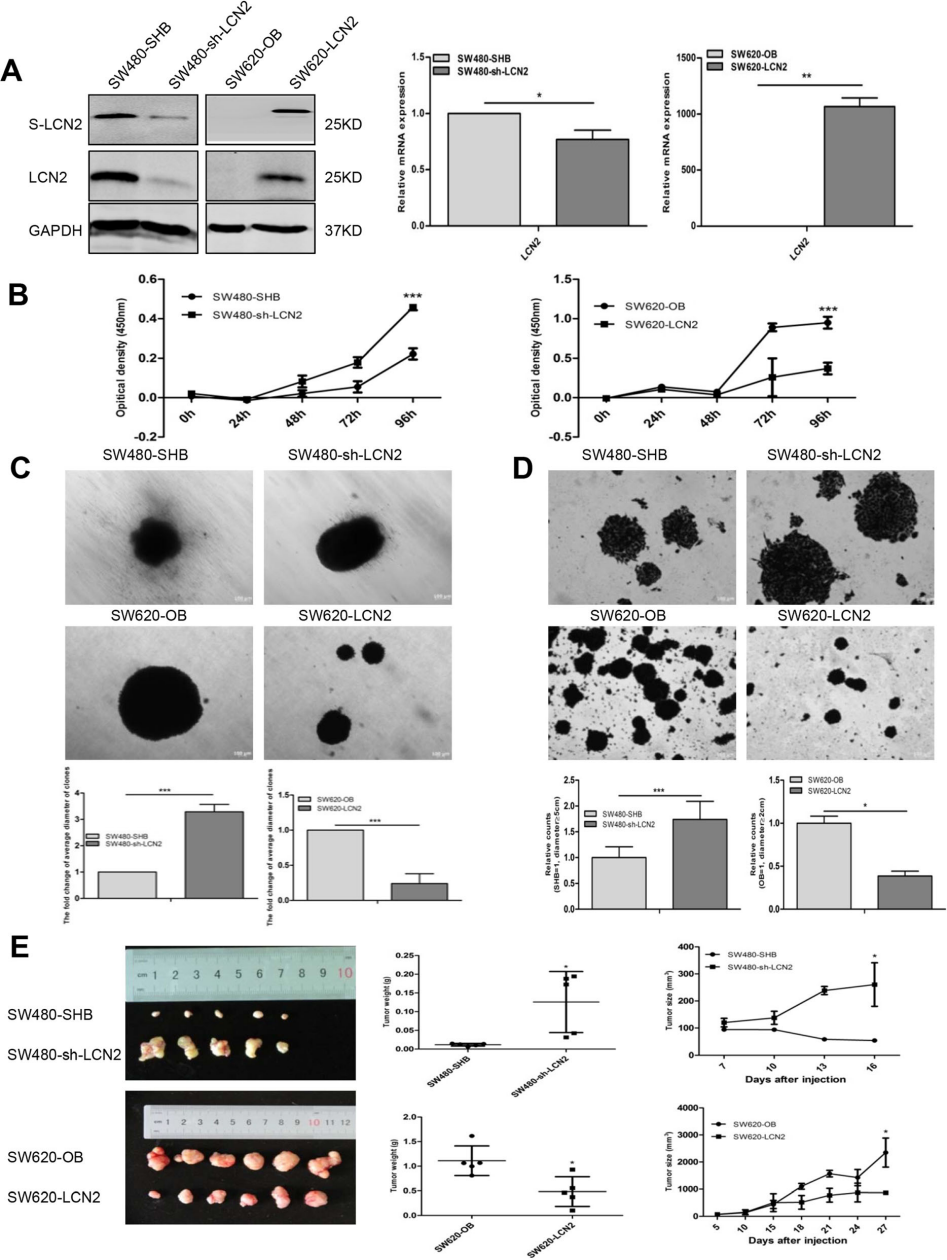
Negative effects of LCN2 on proliferation and tumorigenicity. **a** LCN2 expression confirmed by western blots and real-time PCR in the indicated cell. (s: supernatant) and quantification of relative mRNA expression of LCN2 expression of corresponding cells. **b** Cell proliferation measured by the CCK8 assay, and quantification of OD value at 450 nm of corresponding cells with microplate reader (5000 cells/well, mean ± SD from at least three independent experiments). (C,D) Colony-formation assays on soft agar (**c**) and plate (**d**) with corresponding cells and quantification of the fold change of average clone diameter of eight fields (for soft agar colony-formation assay) and relative counts based on the colony diameter (for plate colony-formation assay, SW480-SHB/sh-LCN2: diameter ≧5 cm; SW620-OB/LCN2: diameter ≧2 cm) (mean ± SD from at least three independent experiments. Scale bars, 100 μm). **e** Tumor weights (**g**) and volumes (mm^3^) of mouse xenografts formed with corresponding cells. SW620-OB (5 × 10^6^), SW620-LCN2 (5 × 10^6^), SW480-SHB (1 × 10^7^), and SW480-sh-LCN2 (1 × 10^7^) cells in 100 μl PBS were inoculated subcutaneously into the left shoulders of 4 to 5-week-old BALB/c nude mice. Tumor length and width were measured with calipers, and tumor volumes were calculated according to the equation: V (mm^3^) = ½ [width^2^ (mm^2^) × length (mm)]. Growth curves were plotted based on mean tumor volume within each experimental group at the indicated time points. Tumor growth was observed for 27 days (for SW620-OB and SW620-LCN2 cells) and 16 days (for SW480-SHB and SW480-sh-LCN2 cells), respectively. * *P* < .05, ** *P* < .01, *** *P* < .001

The negative relationship between LCN2 and Ki67 expression in clinical samples (Table 2) led us to explore whether LCN2 expression in CRC cells affected their proliferation or tumorigenicity. We first established SW620 and RKO cells that stably expressed LCN2 (designated as SW620-LCN2 and RKO-LCN2; vector control-OB), and SW480 and HT29 cells that LCN2 was knocked down (designated as SW480-sh-LCN2 and HT29-sh-LCN2; vector control-SHB) (Fig. 2a, Additional file 4: Figure S1A).

In vitro growth kinetics assays showed that LCN2-knockdown significantly promoted cell proliferation, while the proliferation rate was lower in LCN2-overexpressing cells than in control cells (Fig. 2b, Additional file 4: Figure S1B). Furthermore, LCN2 overexpression effectively reduced clonogenicity, while LCN2 knockdown cells formed bigger colonies than corresponding control cells in both soft agar and plate colony-formation assays (Fig. 2c, d, Additional file 4: Figure S1C,D). Next, to confirm the effect of LCN2 on growth in vivo, four groups of BALB/c nude mice were implanted subcutaneously with SW620-LCN2 cells, SW480-sh-LCN2 cells and corresponding control cells (SW620-OB or SW480-SHB) on the left shoulders. As expected, tumors from SW620-LCN2 cells had dramatically lower volumes and weights than those from SW620-OB cells, while SW480-sh-LCN2 cells formed larger tumor masses and proliferated much faster than SW480-SHB cells (Fig. 2e). In addition, the elevated Ki67 (one of the classic proliferation markers) expression in SW480-sh-LCN2 tumors and decreased Ki67 expression in SW620-LCN2 tumors (Additional file 5: Figure S2A) were consistent with the negative correlation between LCN2 and Ki67 expression in clinical samples (Table 2).

### LCN2 reverses epithelial-mesenchymal transition in CRC

The IHC staining results of E-cadherin and Vimentin in tumor xenografts formed by injecting SW480-SHB, SW480-sh-LCN2, SW620-OB and SW620-LCN2 cells subcutaneously showed that E-cadherin expression was reduced in LCN2-knockdown but increased in LCN2-overexpression mouse xenograft tissue compared to the corresponding control xenograft tissue. While Vimentin expression was elevated in LCN2-knockdown but declined in LCN2-overexpression mouse xenograft tissue compared to the corresponding control xenograft tissue (Additional file 5: Figure S2A).

**Fig. 3:**
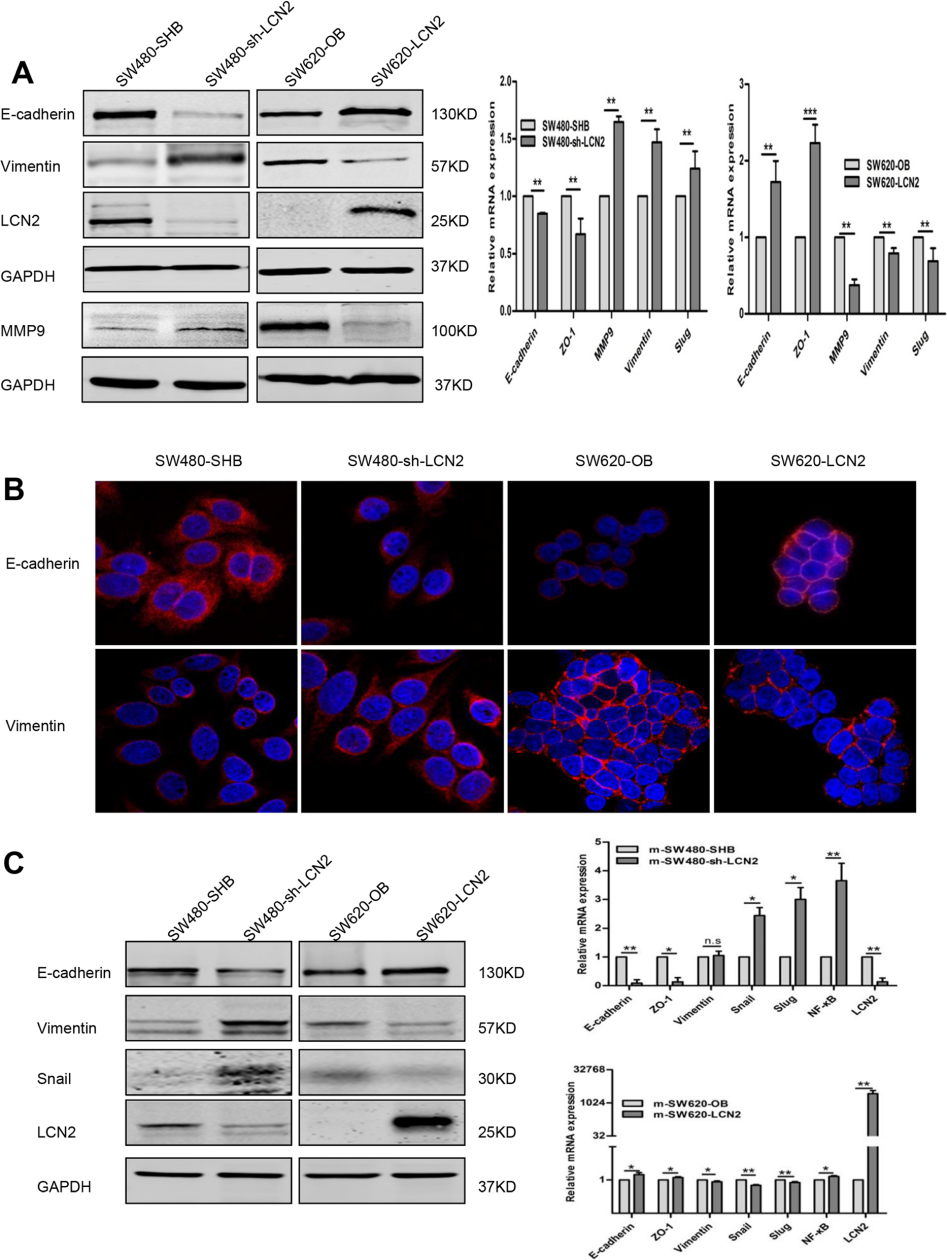
LCN2 inhibits EMT characteristics in vitro and in vivo. **a** Western blots and real-time PCR analysis of EMT markers on LCN2 overexpression in SW620 or knockdown in SW480 cells, and quantification of relative mRNA expression of EMT markers and key proteins of corresponding cells (mean ± SD from at least three independent experiments). **b** Immunofluorescent staining of E-cadherin and Vimentin in corresponding cells. **c** Western blots and real-time PCR analysis of EMT characteristics in mouse xenografts (mean ± SD from at least three independent experiments). * *P* < .05, ** *P* < .01, scale bars, 100 μm

Furthermore, LCN2 overexpression in SW620 and RKO cells significantly inhibited the expression of Vimentin and MMP9 but increased the expression of E-cadherin in protein level. LCN2 also promoted E-cadherin and ZO-1 expression and blocked MMP9, vimentin and slug expression by detection of real-time PCR (Fig. 3a, Additional file 5: Figure S2B,C). The simultaneous upregulation of Vimentin and MMP9 and downregulation of E-cadherin were also found in LCN2-knockdown cells (SW480 and HT29) in protein level, and mRNA detection showed similar results (Fig. 3a, Additional file 5: Figure S2B,C). Moreover, we also observed that EMT-like morphological features, such as a spindle-shaped, fibroblast-like appearance, were induced by knockdown of LCN2 (Additional file 6: Figure S3A), and E-cadherin was lost from cell-to-cell contacts while Vimentin was increased, as assessed by immunofluorescence (Fig. 3b). Likewise, overexpression of LCN2 induced a cuboid, clustered morphology (Additional file 6: Figure S3B) and increased E-cadherin expression without influencing its location and decreased Vimentin expression, as assessed by immunofluorescence (Fig. 3b). Consistent with the in vitro results, similar changes in EMT markers of both protein and mRNA levels were found in mouse xenografts (Fig. 3c).

In summary, these findings revealed that LCN2 was of critical importance in regulating the EMT-MET plasticity of CRC.

### LCN2-induced suppression of metastasis and invasion in CRC

**Fig. 4:**
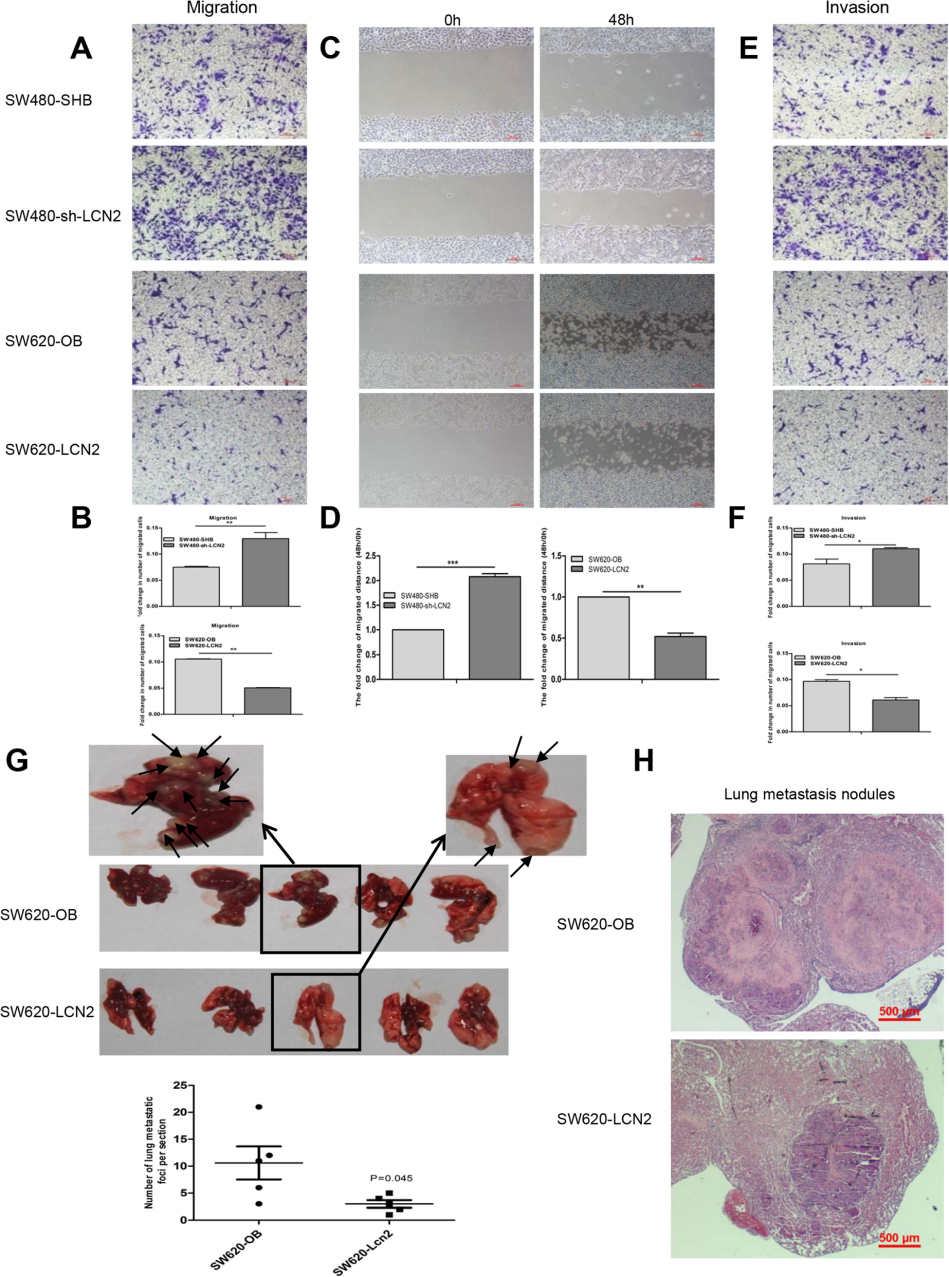
LCN2-mediated inhibition of metastasis/invasion in CRC. **a**, **b** Migration assay of corresponding cells and quantification of cell migration was measured with microplate reader at 570 nm of OD value. (*n* = 3, mean ± SD, scale bars, 100 μm). (C,D) Wound-closure assays of LCN2-overexpressing and LCN2-knockdown cells and corresponding vector control cells and quantification of the fold change of average migrated distance (48 h/0 h) of wound healing assays (*n* = 3, mean ± SD, scale bars, 100 μm). **e**, **f** Invasion assay of corresponding cells and quantification of cell invasion was measured with microplate reader at 570 nm of OD value. (*n* = 3, mean ± SD, scale bars, 100 μm). **g** Metastatic tumor nodules in the lungs of nude mice after injecting LCN2-overexpressing cells and control cells and quantification of the number of lung metastatic foci per section (*n* = 5 mice/group, *P* = .043). **h** Hematoxylin and Eosin (H.E.) staining of lung metastases of nude mice with corresponding cells (scale bars, 500 μm). * *P* < .05, ** *P* < .01, *** *P* < .001

During cancer progression, EMT contributes to invasiveness [22]. Since LCN2 regulated the expression of EMT markers in CRC cells, we hypothesized that LCN2 was also involved in the progression of metastasis and invasion. Indeed, LCN2-knockdown cells migrated through the membrane to a greater extent (Fig. 4a, b and Additional file 7: Figure S4A,B) and the wound closure was significantly greater than in control cells (Fig. 4c, d and Additional file 7: Figure S4C,D). Furthermore, knockdown of LCN2 provoked a greater degree of invasion through Matrigel-coated membranes (Fig. 4e, f and Additional file 7: Figure S4E,F). Against these, the overexpression of LCN2 dramatically reduced the migration, invasiveness, and motility of CRC cells (Fig. 4a-f and Additional file 7: Figure S4), implying that LCN2 mitigated migration/invasion in CRCs in vitro.

To further investigate the role of LCN2 in lung metastasis in vivo, SW620-LCN2 and SW620-OB cells were injected into tail vein of nude mice, followed by assessment of metastatic nodules in the lungs. The number of lung metastatic tumors in the nude mice with SW620-OB cells was more than that in the nude mice with SW620-LCN2 cells (Fig. 4g, h).

Taken together, these results demonstrated that LCN2 strongly inhibited migratory and invasive behaviors in CRCs.

### LCN2 blocks NF-κB signaling pathway and NF-κB promoter activity

**Fig. 5:**
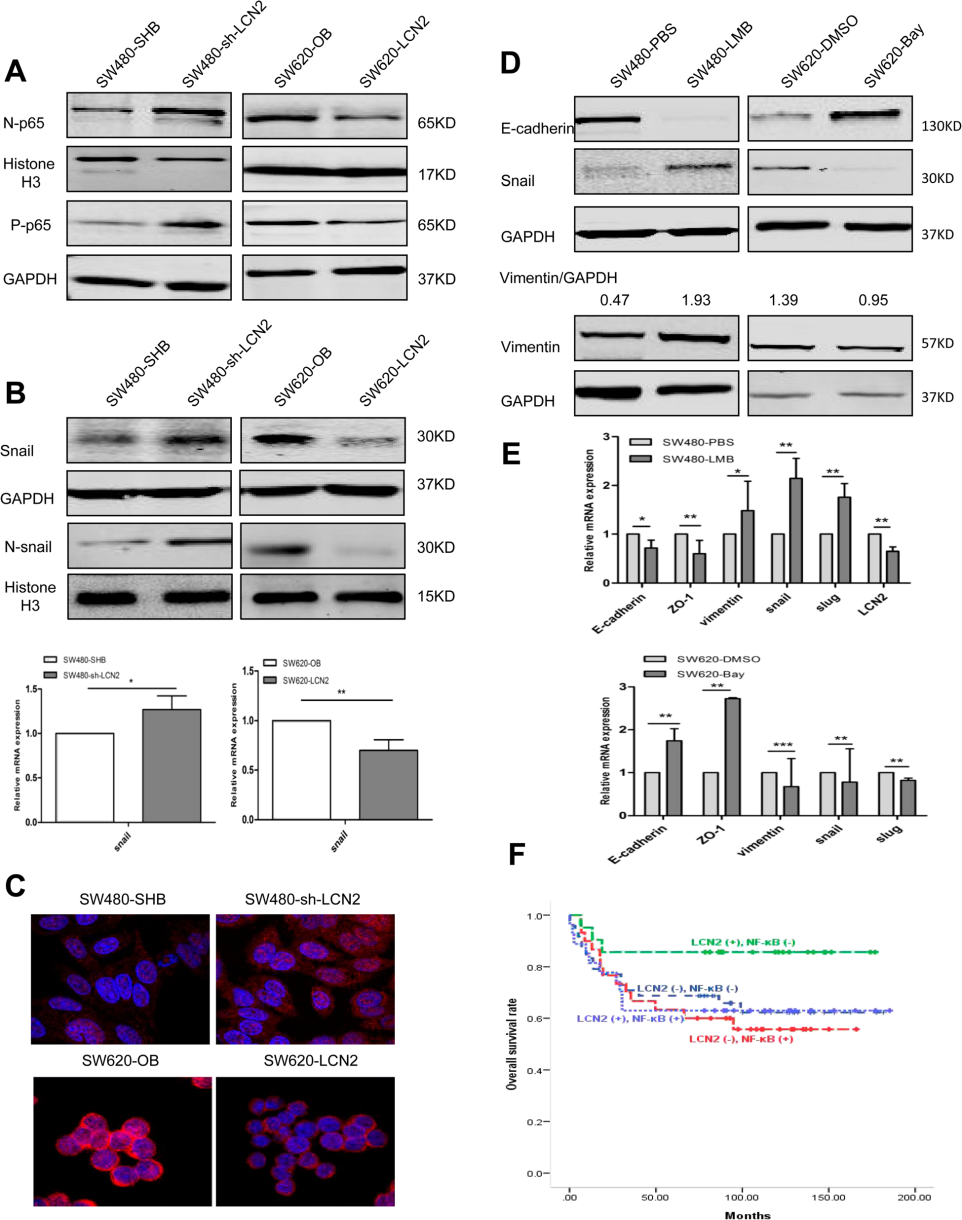
LCN2 suppresses the NF-κB/snail signaling pathway and attenuates NF-κB promoter activity. **a** Western blots detection of p65 expression in LCN2-overexpressing and LCN2-knockdown cells. **b** Western blots detection, quantification of relative mRNA expression of snail expression in corresponding cells. **c** Immunofluorescence of snail expression and location in the indicated cells. **d**, **e** Western blots detection of EMT markers and snail changes (**d**) and quantification of relative mRNA expression of EMT markers (**e**) after treatment with LMB (20nM for 10 h) and Bay11-7082 (50 μM for 3 h) in corresponding cells. The grey value ratio of Vimentin/GAPDH was shown. **f** Survival analysis (Kaplan-Meier method, log-rank test) of combined LCN2 and NF-κB expression in CRC patients (*n* = 126; group 1 (*n* = 21, 3 died) *vs* group 2 (*n* = 48, 17 died), *P* = .083; group 1 *vs* group 3 (*n* = 27, 10 died), *P* = .088; group 1 *vs* group 4 (*n* = 30, 13 died), *P* = .042). Values shown in real-time PCR assay are the mean ± SD from at least three independent experiments. (N, nuclear; P-phosphorylated). * *P* < .05, ** *P* < .01

Since the NF-κB/snail signaling pathway is known to enhance the invasiveness and metastatic properties of several types of cancer cells [17, 18], we sought to investigate the association between this pathway and LCN2 expression. We first assessed the expression of NF-κBp65 (p65) and snail in LCN2-knockdown and LCN2-overexpressing cells in protein and mRNA levels, and found that knockdown of LCN2 significantly elevated the expression of phosphorylated p65 (p-p65) and the nuclear accumulation of both p65 and snail in both SW480 and HT29 cells (Fig. 5a, b, c and Additional file 8: Figure S5A,B), while overexpression of LCN2 remarkably inhibited p-p65 expression and the nuclear accumulation of both p65 and snail in both SW620 and RKO cells (Fig. 5a, b, c and Additional file 8: Figure S5C,D).

Interestingly, a luciferase promoter assay showed that LCN2 effectively attenuated the promoter activity of NF-κB (Additional file 9: Figure S6A,) and NF-ĸB was confirmed here to promote both snail and Vimentin expression and reduce E-cadherin expression in protein level of CRC cells by using Leptomycin B (LMB, the activator of NF-κB) or Bay11-7082 and JSH-23 (the specific inhibitor of NF-κB). Results of real-time PCR also showed that NF-ĸB inhibited E-cadherin and ZO-1 expression and promoted Vimentin, snail and slug expression (Fig. 5d, e and Additional file 9: Figure S6B,C,D). These results suggested that LCN2 inhibited the NF-κB signaling pathway and its promoter activity.

Next, to investigate the relationship between LCN2/NF-κBp65 expression and the prognosis of CRC patients, we divided the clinical samples into four groups according to LCN2 and nuclear NF-κBp65 expression: 1: LCN2 (+)/NF-κBp65 (−), *n* = 21, 3 died; 2: LCN2 (−)/NF-κBp65 (−), *n* = 48, 17died; 3: LCN2 (+)/NF-κBp65 (+), *n* = 27, 10 died; 4: LCN2 (−)/NF-κBp65 (+), *n* = 30, 13 died (follow-up time, 1–200 months; median, 52 months). The results showed that patients with LCN2 (+)/NF-κBp65 (−) expression had more favorable overall survival than those with LCN2 (−)/NF-κBp65 (+) expression (Fig. 5f, *P* = 0.042). The 5-year survival rate of group 1 (LCN2 (+)/NF-κBp65 (−), 86%) was higher than that of group 4 (LCN2 (−)/NF-κBp65 (+), 60%). However, no significant difference (*P* = 0.237) was found between LCN2 (+) and LCN2 (−) patients (Additional file 9: Figure S7E).

### LCN2 blocks EMT and migration by targeting the NF-κB/snail signaling pathway

**Fig. 6:**
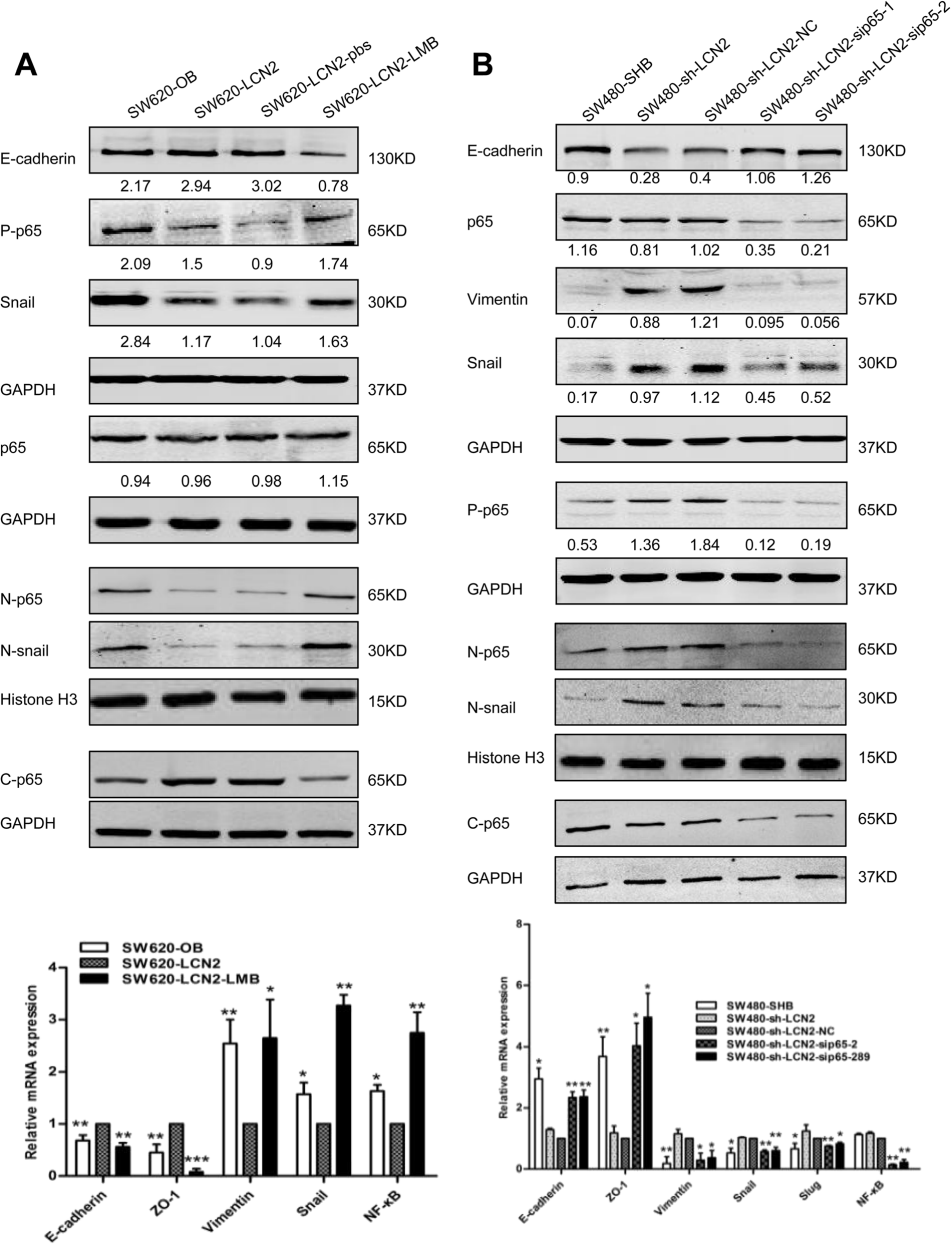
EMT changes depend on the LCN2/NF-κB/Snail pathway. **a**, **b** Changes of EMT markers measured by western blots and real-time PCR after enhancing NF-ĸB activity with LMB (40nM for 6 h), and quantification of relative mRNA expression of EMT markers in SW620-LCN2 cells (**a**) and suppressing NF-κB activity with specific siRNA (50nM for 48 h), and quantification of relative mRNA expression of EMT markers in SW480-sh-LCN2 cells (**b**), The grey value ratios of the corresponding proteins/GAPDH were shown. Values shown in real-time PCR assay are the mean ± SD from at least three independent experiments. (N, nuclear; C, cytoplasm; P, phosphorylated; NC, negative control). * *P* < .05, ** *P* < .01, *** *P* < .001

In clinical specimens, the nuclear snail was less frequently expressed in patients with higher LCN2 and negative NF-κB (*P* = 0.045) (Additional file 9: Figure S6F). We then hypothesized that the NF-κB/snail signaling pathway was involved in LCN2-inhibited EMT and metastasis in CRC. Next, we restored the decreased p65 expression using LMB in SW620-LCN2 and RKO-LCN2 cells and attenuated the enhanced p65 expression with specific siRNA in SW480-sh-LCN2 and HT29-sh-LCN2 cells, following by assessing the changes in EMT markers. As expected, LMB treatment in LCN2-overexpression cells enhanced p65 and snail nuclear expression together with phosphorylated p65 expression and blocked the increase of E-cadherin and the decrease of snail expression in protein level, and inhibited the upregulated E-cadherin and ZO-1 and the downregulated Vimentin and snail expression in mRNA level in SW620-LCN2 cells (Fig. 6a). Furthermore, detection of protein level in RKO-LCN2 cells showed that LMB also restored the decrease of MMP9 and snail expression in protein level, and the increase of ZO-1 and the decrease of MMP9, snail and slug expression in mRNA level (Additional file 10: Figure S7A).

Moreover, transfection of siRNA for p65 significantly inhibited nuclear accumulation of p65 and snail, and reversed the EMT changes in SW480-sh-LCN2 and HT29-sh-LCN2 cells in both protein and mRNA levels (Fig. 6b, Additional file 10: Figure S7B).

**Fig. 7:**
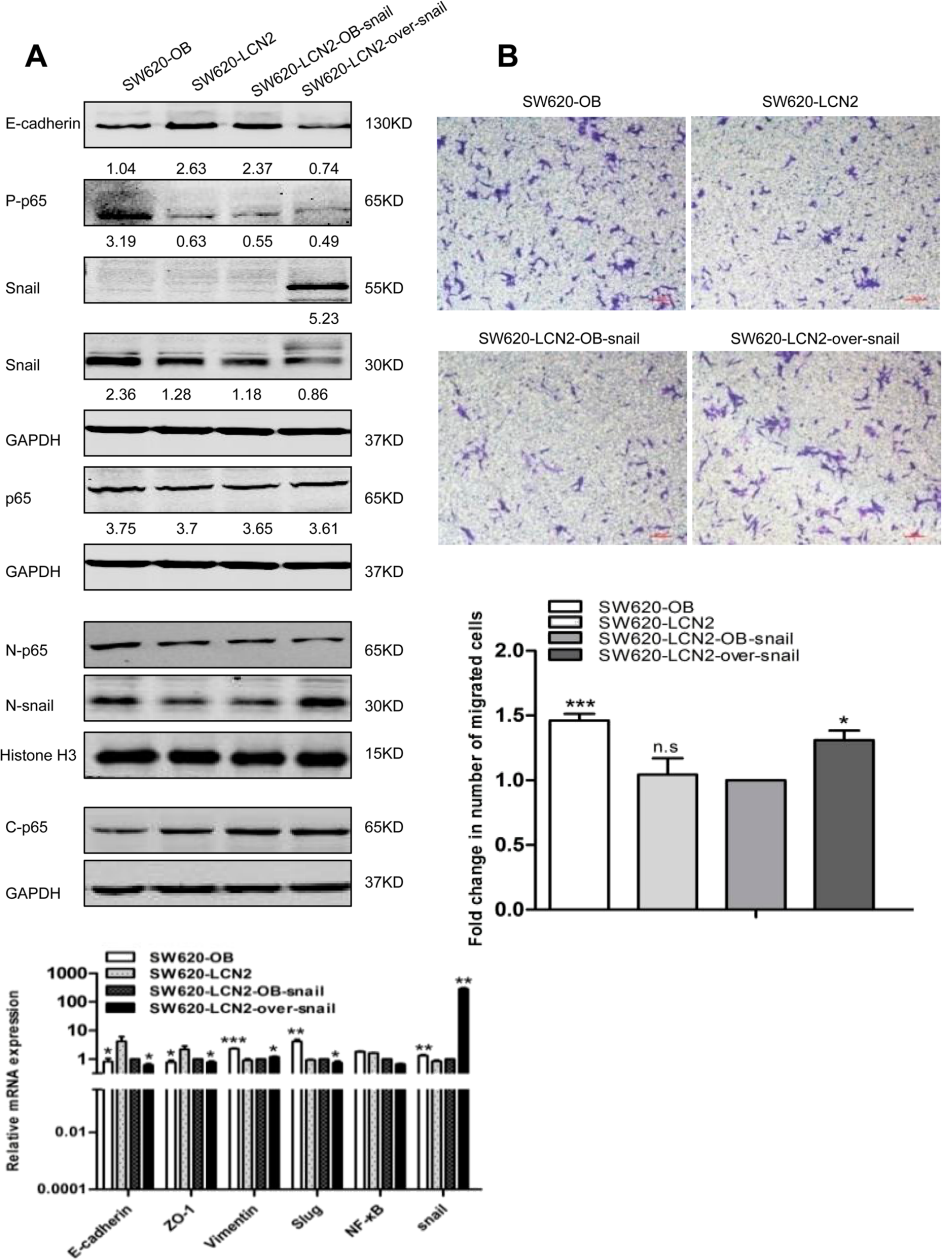
EMT changes depend on the LCN2/NF-κB/Snail pathway. **a** Changes of EMT markers measured by western blots and real-time PCR after restoring snail expression, and quantification of relative mRNA expression of EMT markers in SW620-LCN2 cells. Snail protein (55KD) is a fusion protein with green fluorescence protein (GFP). The grey value ratios of the corresponding proteins/GAPDH were shown. **b** Migration assay and quantification of migration after overexpression of snail in SW620-LCN2 cells (OD values were measured by a microplate reader at 570 nm). Values shown in real-time PCR assay are the mean ± SD from at least three independent experiments. (N, nuclear; C, cytoplasm; P, phosphorylated; over, overexpression). * *P* < .05, ** *P* < .01, *** *P* < .001. Scale bars, 100 μm

Furthermore, restoring snail levels by transfection with snail-overexpression vector also effectively eliminated the increase in E-cadherin of SW620-LCN2 cells, and the decrease in MMP9 expression of RKO-LCN2 cells (no E-cadherin expression was detected) in protein level. Moreover, mRNA results also showed snail overexpression reversed the upregulated E-cadherin and ZO-1 expression, as well as the downregulated expression of Vimentin and slug expression in SW620-LCN2 cells (Fig. 7a), and blocked the increase of ZO-1, and the decrease of MMP9 and slug expression in RKO-LCN2 cells (Additional file 11: Figure S8A).

Furthermore, the decreased migration ability induced by LCN2 overexpression in both SW620 and RKO cells was also abrogated by snail-overexpression (Fig. 7b, Additional file 11: Figure S8B).

Since many researches have reported NF-κB could induce snail promoter activity [23–25], our results suggested a novel mechanism that LCN2 suppressed metastasis in CRC cells by inhibiting the NF-κB-dependent activation of snail and EMT.

## Discussion

Here, we provide new evidence directly showing that LCN2 prevents CRC cells from undergoing EMT and metastasis/invasion by targeting the promoter activity of NF-κB, which inhibits the NF-κB/snail signaling pathway. These findings identify the LCN2/NF-κB/snail signaling pathway as highly suitable candidates for therapeutic agents for CRC patients.

It has been reported that LCN2 inhibits tumor metastasis/invasion in diverse cancers [5, 8, 26]. Here, we identified two aspects of the clinical importance of LCN2 in CRC. First, patients with higher LCN2 and negative NF-κB had a better prognosis. Second, immunohistochemical analysis of CRC tissues also showed that LCN2 expression was positively correlated with E-cadherin expression and negatively associated with nuclear-β-catenin. Thus, we conclude that LCN2 is an important modulator in the progression of CRC.

Our data showed that LCN2 was higher in CRC tissues than in normal tissues or metastatic lymph node lesions, consistent with the TCGA database and with a previous gene expression profiling study, which showed that LCN2 is highly upregulated in CRC tissues [27]. Elevated levels of serum LCN2 have also been observed in our data. Although the regulatory actions of elevated LCN2 in cancer are not yet fully understood, aberrant LCN2 expression has been found under several conditions. Recent studies have reported that LCN2 expression is elevated in both the colorectal adenoma-carcinoma sequence and cancer progression [28]. Furthermore, the LCN2 promoter non-methylated status (67.2%) is much higher than its methylated status (32.8%) in breast cancer patients [29]. In bladder cancer, the LCN2 methylation ratio of the CpG site on the promoter is also much lower in the tumor tissue than in the normal tissue [30].

Moreover, inflammatory cytokines, such as interleukin (IL)-6, induce LCN2 expression depending on activation of the STAT3 signaling pathway, IL-1β and the tumor cell-derived factor IL-10 also promote LCN2 expression in lung [31] and breast cancer [32]. Interestingly, upregulated expression of IL-1β, IL-6, and TNF-α, and sustained activation of the STAT1, STAT3, and JNK pathways, both occur in LCN2-knockout mice along with an increase in the susceptibility to infection with *Klebsiella pneumoniae* or *Escherichia coli* in the liver and in mice with breast cancer [33, 34]. It has been noted that inflammation of the colon, including inflammatory bowel disease (IBD) or colitis, increases the risk of CRC by causing a cellular immune response and accumulating genetic alterations that might trigger specific oncogenic pathways [35, 36]. Besides, the finding that the long-term prognosis of CRC is poorer in patients with IBD than in those with sporadic CRC [37] implies that elevated LCN2 expression plays a prominent role in CRC progression.

LCN2 has been implicated in the regulation of proliferation in terms of its association with a variety of proliferative cells [38]. On one hand, LCN2 expression provokes tumor growth and progress in breast cancer [39] and increases the migration/invasion of pancreatic cancer [3]. On the other hand, LCN2 leads to apoptosis in leukemia cells [40] and acts as a suppressor of proliferation and metastasis in hepatocellular carcinoma [5, 7]. In our study, LCN2 inhibited the proliferation and metastasis/invasion of CRC cells in vitro, as well as tumor growth and lung metastasis in vivo. This is consistent with previous reports that LCN2 suppresses invasion and the liver metastasis of highly metastatic CRC KM12SM cells [41]. Although the involvement of the NF-κB/snail signaling pathway in promoting metastasis/invasion through EMT has been shown in vivo and in vitro [23, 42], the regulation of LCN2 in the NF-κB/snail signaling pathway-induced metastasis and EMT in cancer had not been reported.

It is widely accepted that NF-κB induces a significant increase in the expression level of snail [18, 23, 25], which leads to a remarkable decrease of E-cadherin-mediated intracellular adhesion, subsequently inducing EMT and metastasis/invasion in cancer cells. We tested the hypothesis that LCN2 blocks the NF-κB/snail signaling pathway. Indeed, our results revealed that LCN2 significantly decreased the translocation of p65 into the nucleus by suppressing NF-κB promoter activity, leading to inhibition of the snail-dependent EMT and migration in CRC. Although many studies have shown that NF-κB enhances LCN2 expression to promote progression in a series of cancers [9, 43], we focused on the regulation of LCN2 in NF-κB/snail pathway-induced cancer progression. The results showed that LCN2 may indirectly regulate NF-κB activity, but the specific mechanisms underlying their interactions in different cancers need further exploration. These results provide a new perspective on the possible underlying mechanism by which LCN2 is involved in metastasis and EMT progression in cancer. Moreover, less nuclear staining of snail was found in the patients with higher LCN2 and negative NF-κB. Therefore, expression of LCN2 combined with NF-κB may be a candidate biomarker for CRC patients.

Collectively, these results clearly demonstrate that LCN2 suppresses proliferation, metastasis/invasion, and EMT by attenuating the promoter activity of NF-κB in CRC, which inhibits the NF-κB/snail signaling pathway. Thus, LCN2 may play a protective role against EMT and metastasis in CRC.

## Conclusions

Overall, our results identify LCN2 exhibits a strong tumor-suppressive effect by acting as a new upstream inhibitor of the NF-κB/snail pathway to control colorectal cancer progression, and suggest promising applications for LCN2/NF-ĸB/snail pathway as novel prognostic, and therapeutic strategies for colorectal cancer.

## Additional files

Additional file 1: Si-RNA sequences. (DOCX 12 kb)
Additional file 2: List of Primary Antibodies for Western blot, Immunohistochemistry and Immunofluorescence. (DOCX 13 kb)
Additional file 3: Primer Sequences and Product Length. (DOCX 13 kb)
Additional file 4: Figure S1. Changes of proliferation and colony-formation capacity in the indicated cells. (A) Western blots detection of LCN2 expression in LCN2 knockdown cells (HT29) and LCN2 overexpressing cells (RKO). (B) Quantification of CCK8 measurement (OD value) of proliferation 96 h after indicated cells were seeded in 96-well plates (2500 cells/well, measured with microplate reader at 450 nm). (C, D) Colony formation assays on soft agar and quantification of the fold change of average diameter (C) and plates and quantification of relative counts based on colony diameter (HT29-SHB/sh-LCN2: diameter ≧7 cm; RKO-OB/LCN2: diameter ≧2.5 cm) with corresponding cells (D). Values shown are the mean ± SD from at least three independent experiments. * *P* < .05, ** *P* < .01, *** *P* < .001, scale bars, 100 μm. (TIF 4350 kb)
Additional file 5: Figure S2. EMT marker expression in the indicated cells and tumors. (A) Immunohistochemical staining of E-cadherin, Vimentin and Ki67 and and H.E. staining of tumor masses formed by subcutaneous injecting SW480-sh-LCN2, SW620-LCN2, and corresponding control cells into the left shoulders of nude mice. (B,C) Western blot and real-time PCR assays and quantification of relative mRNA expression of EMT marker changes in an LCN2-knockdown cell line (HT29) and an LCN2-overexpressing cell line (RKO). Values shown are the mean ± SD from at least three independent experiments. * *P* < .05, ** *P* < .01, *** *P* < .001. Scale bars, 100 μm. (TIF 5184 kb)
Additional file 6: Figure S3. (A) Morphology changes of LCN2-knockdown cells (SW480 and HT29) and corresponding control cells. (B) Morphology changes of LCN2-overexpression cells (SW620 and RKO) and corresponding control cells. Scale bars, 100 μm. (TIF 6239 kb)
Additional file 7: Figure S4. Migration, invasiveness and motility changes in the indicated cells. (A,B) Migration and quantification of the fold change of OD value at 570 nm (measured with a microplate reader) of the indicated cells. (C,D) Motility changes in HT29-sh-LCN2, RKO-LCN2, and control cells and quantification of the fold change of average migrated distance (48 h/0 h) of corresponding cells. (E,F) Invasion and quantification of the fold change of OD value in the indicated cells. Values shown are the mean ± SD from at least three independent experiments. ** *P* < .01, *** *P* < .001. Scale bars, 100 μm. (TIF 8849 kb)
Additional file 8: Figure S5. (A,B) Total and cytoplasm expression of p65 in SW480-sh-LCN2, HT29-sh-LCN2 cells and corresponding control cells. (C,D) Changes of p65 and snail expression in SW620-LCN2, RKO-LCN2 and corresponding control cells, (N, nuclear; C-cytoplasm; P, phosphorylated). (TIF 1603 kb)
Additional file 9: Figure S6. (A) Quantification of the fold change of luciferase promoter reporter assays of NF-κB in LCN2-knockdown and LCN2-overexpressing cells in 293FT cells. (B,C) p65 and snail (total, nuclear and cytoplasm) expression of SW480 cells treated with LMB (20nM for 10 h) (B) and SW620 cells treated with Bay11-7082 (50 μM for 3 h) (C). (D) Western blots and quantification of EMT markers, p65 (nuclear and cytoplasm) expression in SW620 cells treated with JSH-23 (50 μM for 1 h), (N, nuclear; C-cytoplasm). (E) Survival analysis (Kaplan-Meier method, log-rank test) of LCN2 expression in CRC patients (*n* = 126, *P* = .237). (F) Correlation between LCN2/NF-κB expression and nuclear snail expression in clinical samples (group 1: LCN2 (+)/NF-κB (−), total number = 21, 7 cases with positive snail; group 2–4: other groups, total number = 103, 59 cases with positive snail). Values shown are the mean ± SD from at least three independent experiments. * *P* < .05, ** *P* < .01, *** *P* < .001. (TIF 2481 kb)
Additional file 10: Figure S7. Association between LCN2 and NF-ĸB/snail pathway in the indicated cells. (A) Changes of MMP9, p65 and snail expression in RKO-LCN2 cells and quantification of relative mRNA expression of EMT key proteins after treatment with LMB (40nM, 6 h) in RKO-LCN2 cells. (B) Changes of EMT key proteins, p65 expression and quantification of relative mRNA expression of EMT key proteins after transfection with specific siRNA (50nM, 48 h) of NF-ĸBp65 in HT-29-sh-LCN2 cells, (N, nuclear; C, cytoplasm; P-phosphorylated). The grey value ratios of the corresponding proteins/GAPDH were shown. Values shown in real-time PCR assay are the mean ± SD from at least three independent experiments. * *P* < .05, ** *P* < .01, *** *P* < .001. (TIF 4190 kb)
Additional file 11: Figure S8. EMT and migration changes depend on the LCN2/NF-κB/Snail pathway in RKO-LCN2 cell lines. (A) Changes of MMP9, snail and p65 expression assessed by western blots and quantification of relative mRNA expression of EMT key proteins after restoring snail expression in RKO-LCN2 cells. Snail protein (55KD) is a fusion protein with green fluorescence protein (GFP). The grey value ratios of the corresponding proteins/GAPDH were shown. (B) Migration assay and quantification of migration at OD value of 570 nm with a microplate reader after overexpression of snail in RKO-LCN2 cells. Values shown in real-time PCR assay are the mean ± SD from at least three independent experiments., (N, nuclear; C, cytoplasm; P-phosphorylated; over, overexpression). * *P* < .05, ** *P* < .01, *** *P* < .001. Scale bars, 100 μm. (TIF 6141 kb)

## Abbreviations

CRC: Colorectal cancer
EMT: Epithelial mesenchymal transition
LCN2: Lipocalin2
LMB: Leptomycin B
NF-κB: Nuclear factor kappa-B
p65: NF-κB p65
p-p65: Phosphorylated p65
SHB/OB: Blank control of shRNA/overexpression vector of LCN2
sh-LCN2: shRNA vector of LCN2
