# Supplementary material for: Lipocalin2 suppresses metastasis of colorectal cancer by attenuating NF-κB-dependent activation of snail and epithelial mesenchymal transition
Source: Mol Cancer. 2016 Dec 3;15:77. doi: 10.1186/s12943-016-0564-9 (PMC5135816; doi:10.1186/s12943-016-0564-9)
Supplement: Additional file 1: — Si-RNA sequences. (DOCX 12 kb) [file 12943_2016_564_MOESM1_ESM.docx]

**Additional File 1: Si-RNA sequences**

|  | **Sense** | **Antisense** |
| --- | --- | --- |
| Negative | UUCUCCGAACGUGUCACGUTT | ACGUGACACGUUCGGAGAATT |
| sip65-1 | GAUCAAUGGCUACACAGGATT | UCCUGUGUAGCCAUUGAUCTT |
| sip65-2 | GGAGCACAGAUACCACCAATT | UUGGUGGUAUCUGUGCUCCTT |
