# Supplementary material for: Lipocalin2 suppresses metastasis of colorectal cancer by attenuating NF-κB-dependent activation of snail and epithelial mesenchymal transition
Source: Mol Cancer. 2016 Dec 3;15:77. doi: 10.1186/s12943-016-0564-9 (PMC5135816; doi:10.1186/s12943-016-0564-9)
Supplement: Additional file 2: — List of Primary Antibodies for Western blot, Immunohistochemistry and Immunofluorescence. (DOCX 13 kb) [file 12943_2016_564_MOESM2_ESM.docx]

**Additional File 2: List of Primary Antibodies for Western blot, Immunohistochemistry and Immunofluorescence**

| **Antibody** | **Concentration for WB** | **Concentration for IHC** | **Concentration for IF** | **Specificity** | **Company** |
| --- | --- | --- | --- | --- | --- |
| E-cadherin | / | 1:200 | / | Mouse monoclonal | Dako |
| β-catenin | / | 1:200 | / | Mouse monoclonal | Maixin |
| Ki67 | / | 1:100 | / | Mouse monoclonal | Dako |
| LCN2 | / | 1:200 | / | Mouse monoclonal | R&D |
| NF-κBp65 | / | 1:200 | / | Mouse monoclonal | Santa Cruz |
| Snail | / | 1:200 | / | Rabbit monoclonal | Cell Signaling Technology |
| E-cadherin | 1:1000 | / | 1:200 | Rabbit monoclonal | Cell Signaling Technology |
| vimentin | 1:1000 | / | 1:100 | Rabbit monoclonal | Cell Signaling Technology |
| snail | 1:1000 | / | 1:100 | Rabbit monoclonal | Cell Signaling Technology |
| NF-κB | 1:2000 | / | 1:50 | Rabbit monoclonal | Cell Signaling Technology |
| LCN2 | 1:1000 | / | / | Rabbit polyclonal | Abcam |
| P-NF-κB-p65 | 1:1000 | / | / | Rabbit polyclonal | Cell Signaling Technology |
| Histone H3 | 1:2000 | / | / | Rabbit monoclonal | Cell Signaling Technology |
| GAPDH | 1:5000 | / | / | Mouse monoclonal | Sigma |
