# Supplementary material for: Lipocalin2 suppresses metastasis of colorectal cancer by attenuating NF-κB-dependent activation of snail and epithelial mesenchymal transition
Source: Mol Cancer. 2016 Dec 3;15:77. doi: 10.1186/s12943-016-0564-9 (PMC5135816; doi:10.1186/s12943-016-0564-9)
Supplement: Additional file 3: — Primer Sequences and Product Length. (DOCX 13 kb) [file 12943_2016_564_MOESM3_ESM.docx]

**Additional File 3:** **Primer Sequences and Product Length**

| **Gene** | **Forward primer (5’-3’)** | **Reverse primer (5’-3’)** | **Melting temperature (℃)** | **Product**  **length (bp)** | |
| --- | --- | --- | --- | --- | --- |
| LCN2 | CTCCACCTCAGACCTGATCC | ACCTTGCCTTCTTTGTCTTTGTTGGA | 60 | | 350 |
| E-cadherin | GCCCCATCAGGCCTCCGTTT | CCGAGATGGGGTTGATAATG | 60 | | 241 |
| ZO-1 | TGGCCACAGCCCGAGGCATAT | GTAAGCGCAGCTCCACAGGC | 60 | | 242 |
| Vimentin | GAGAACTTTGCCGTTGAAGC | GCTTCCTGTAGGTGGCAATC | 60 | | 243 |
| Snail | CTAGGCCCTGGCTGCTACAAG | AGCGGGGACATCCTGAGCA | 60 | | 245 |
| Slug | ATATTCGGACCCACACATTACCT | GCAAATGCTCTGTTGCAGTGA | 60 | | 139 |
| NF-κB | GGGCATGCGCTTCCGCTACA | TCCCCACGCTGCCTTCTTGGA | 59 | | 354 |
| GAPDH | ACCACAGTCCATGCCATCAC | TCCACCACCCTGTTGCTGTA | 60 | | 250 |
