# Supplementary figures and images for: Lipocalin2 suppresses metastasis of colorectal cancer by attenuating NF-κB-dependent activation of snail and epithelial mesenchymal transition
Source: Mol Cancer. 2016 Dec 3;15:77. doi: 10.1186/s12943-016-0564-9 (PMC5135816; doi:10.1186/s12943-016-0564-9)

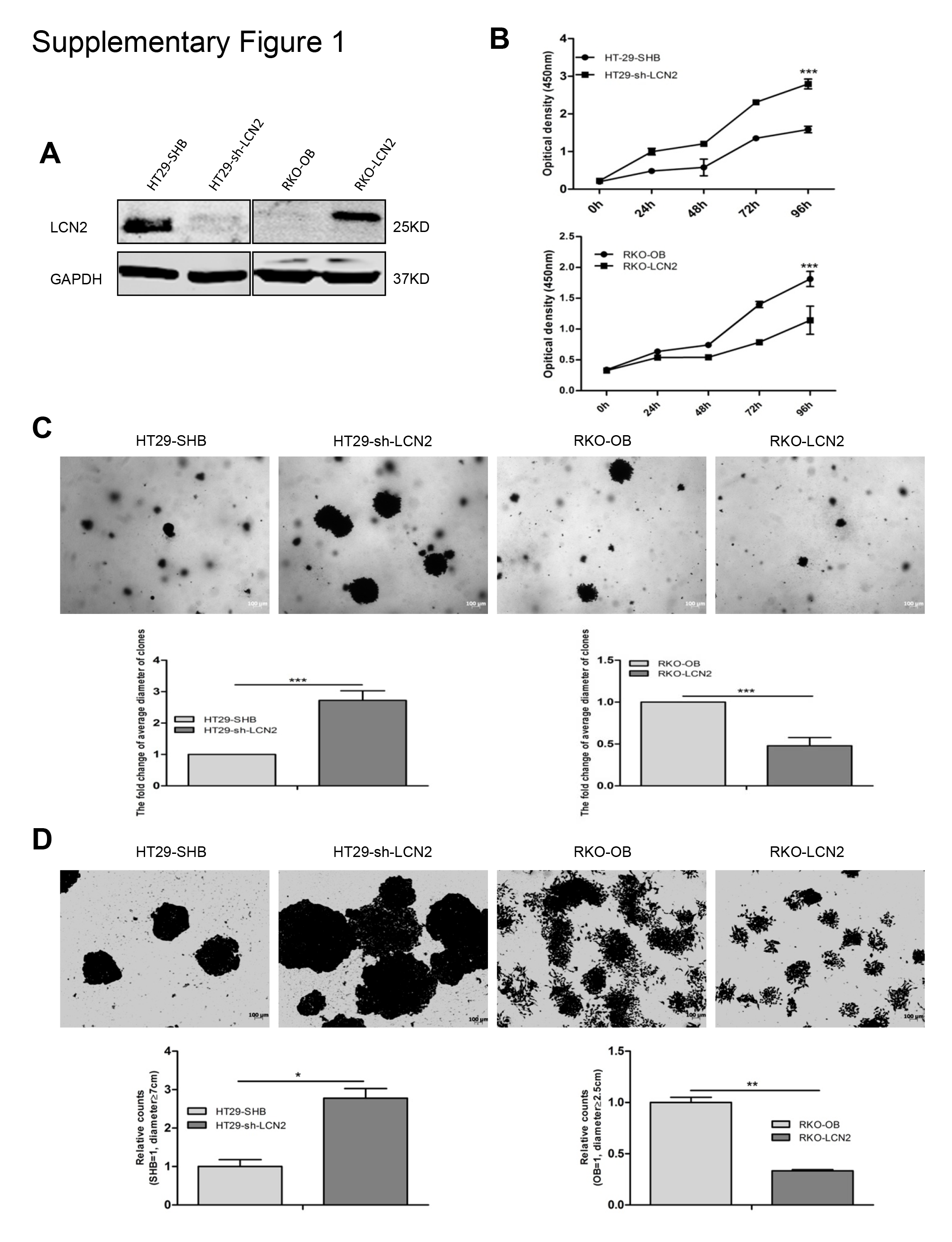

Supplement: Additional file 4: Figure S1. — Changes of proliferation and colony-formation capacity in the indicated cells. (A) Western blots detection of LCN2 expression in LCN2 knockdown cells (HT29) and LCN2 overexpressing cells (RKO). (B) Quantification of CCK8 measurement (OD value) of proliferation 96 h after indicated cells were seeded in 96-well plates (2500 cells/well, measured with microplate reader at 450 nm). (C, D) Colony formation assays on soft agar and quantification of the fold change of average diameter (C) and plates and quantification of relative counts based on colony diameter (HT29-SHB/sh-LCN2: diameter ≧7 cm; RKO-OB/LCN2: diameter ≧2.5 cm) with corresponding cells (D). Values shown are the mean ± SD from at least three independent experiments. * P < .05, ** P < .01, *** P < .001, scale bars, 100 μm. (TIF 4350 kb) [file 12943_2016_564_MOESM4_ESM.tif]

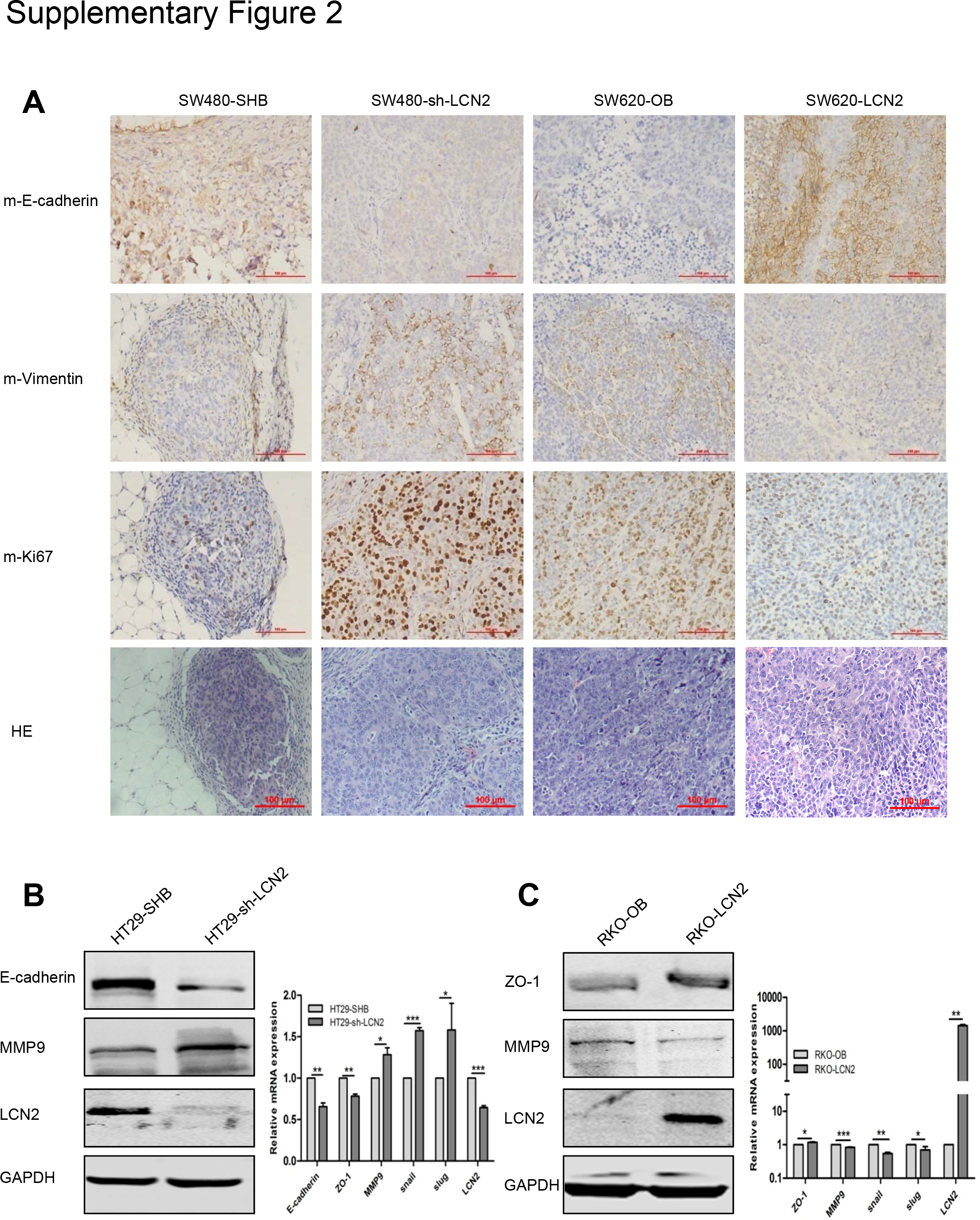

Supplement: Additional file 5: Figure S2. — EMT marker expression in the indicated cells and tumors. (A) Immunohistochemical staining of E-cadherin, Vimentin and Ki67 and and H.E. staining of tumor masses formed by subcutaneous injecting SW480-sh-LCN2, SW620-LCN2, and corresponding control cells into the left shoulders of nude mice. (B,C) Western blot and real-time PCR assays and quantification of relative mRNA expression of EMT marker changes in an LCN2-knockdown cell line (HT29) and an LCN2-overexpressing cell line (RKO). Values shown are the mean ± SD from at least three independent experiments. * P < .05, ** P < .01, *** P < .001. Scale bars, 100 μm. (TIF 5184 kb) [file 12943_2016_564_MOESM5_ESM.tif]

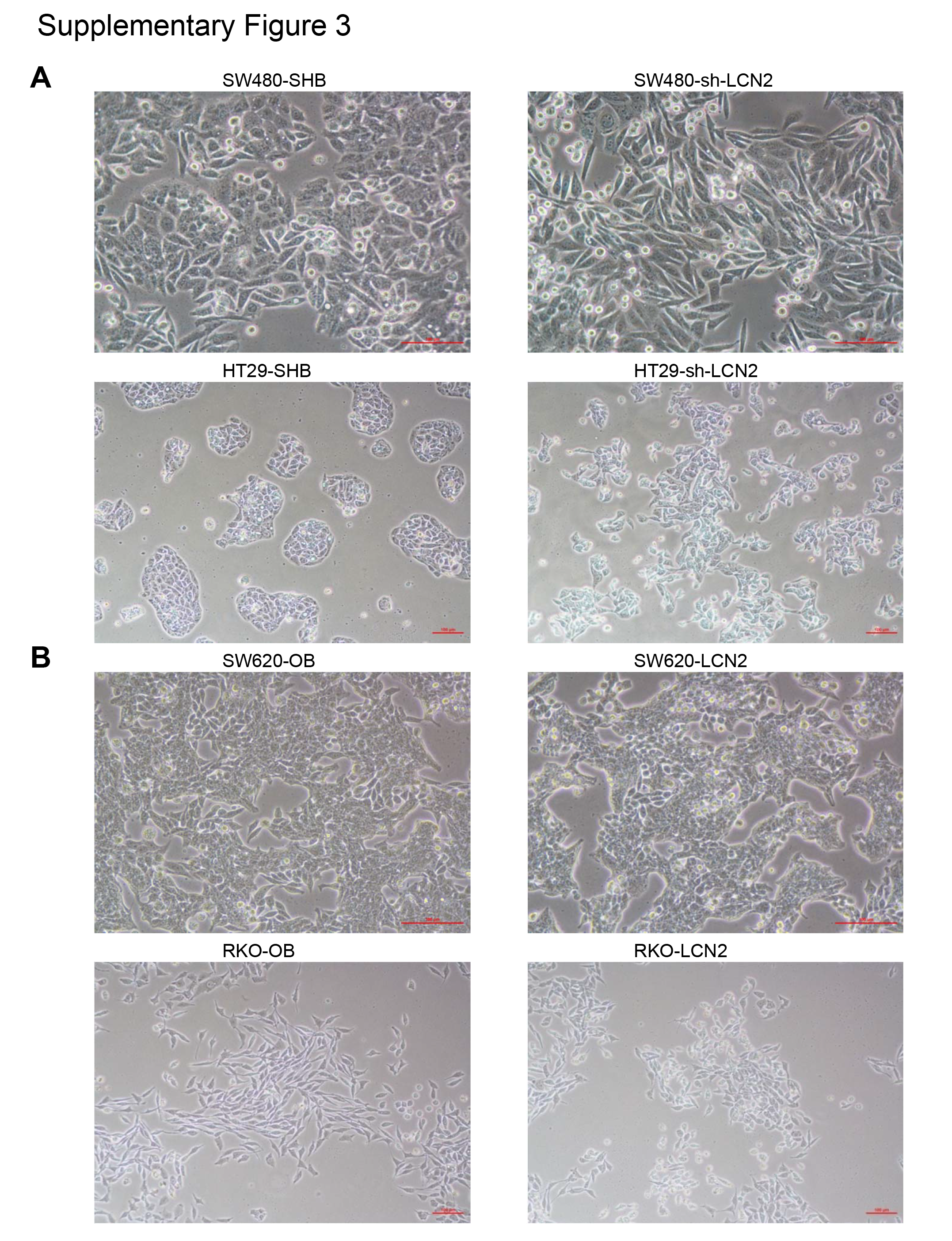

Supplement: Additional file 6: Figure S3. — (A) Morphology changes of LCN2-knockdown cells (SW480 and HT29) and corresponding control cells. (B) Morphology changes of LCN2-overexpression cells (SW620 and RKO) and corresponding control cells. Scale bars, 100 μm. (TIF 6239 kb) [file 12943_2016_564_MOESM6_ESM.tif]

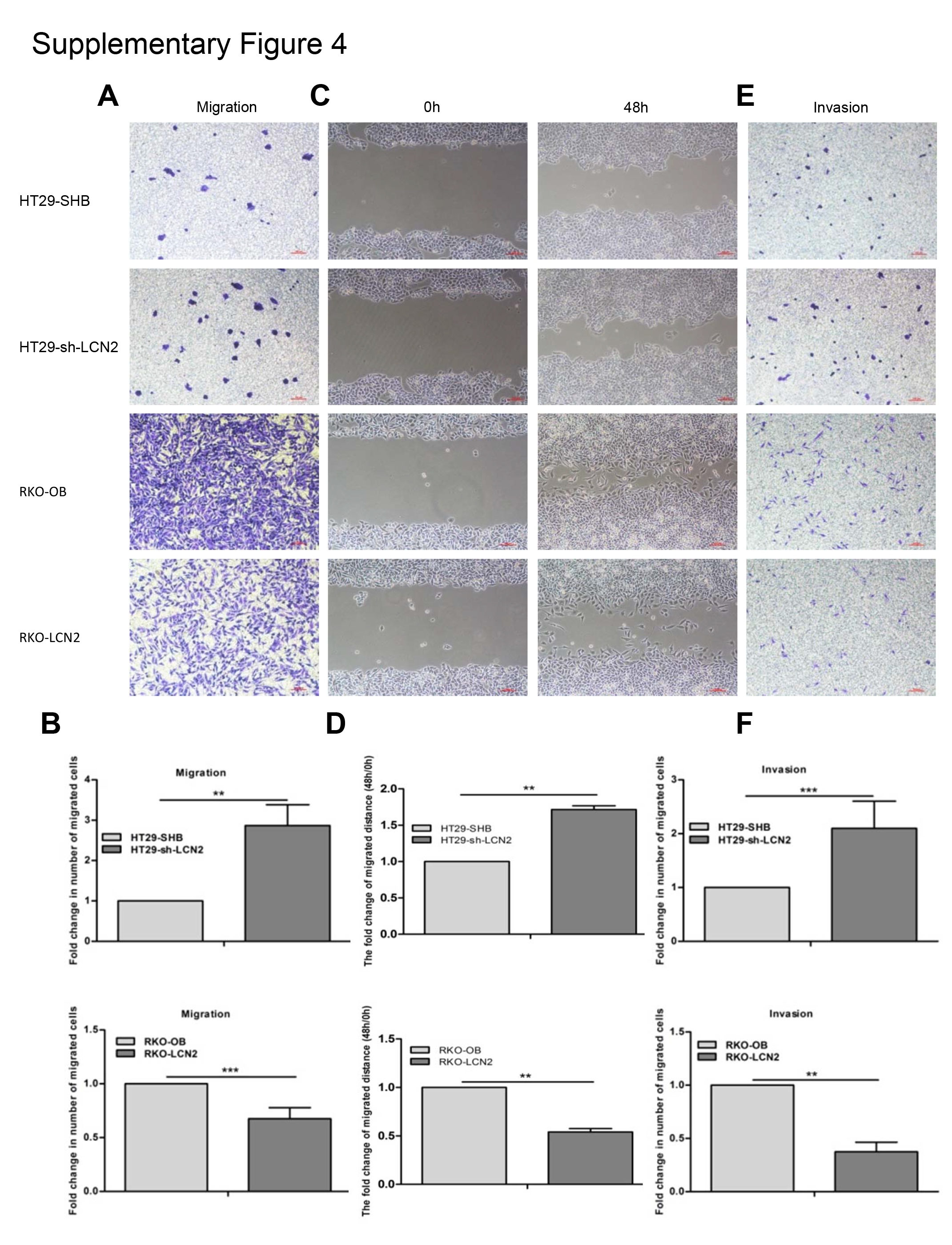

Supplement: Additional file 7: Figure S4. — Migration, invasiveness and motility changes in the indicated cells. (A,B) Migration and quantification of the fold change of OD value at 570 nm (measured with a microplate reader) of the indicated cells. (C,D) Motility changes in HT29-sh-LCN2, RKO-LCN2, and control cells and quantification of the fold change of average migrated distance (48 h/0 h) of corresponding cells. (E,F) Invasion and quantification of the fold change of OD value in the indicated cells. Values shown are the mean ± SD from at least three independent experiments. ** P < .01, *** P < .001. Scale bars, 100 μm. (TIF 8849 kb) [file 12943_2016_564_MOESM7_ESM.tif]

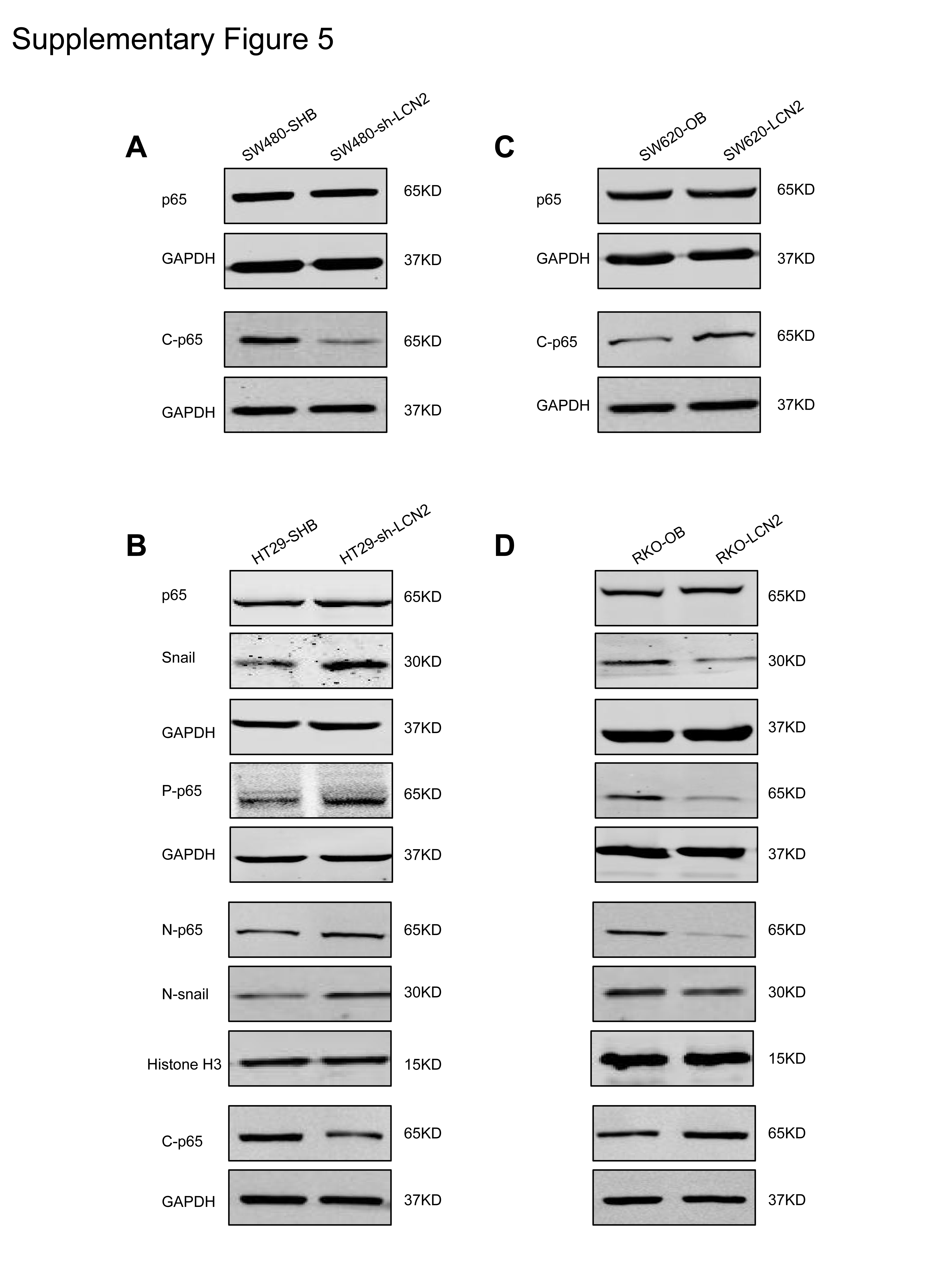

Supplement: Additional file 8: Figure S5. — (A,B) Total and cytoplasm expression of p65 in SW480-sh-LCN2, HT29-sh-LCN2 cells and corresponding control cells. (C,D) Changes of p65 and snail expression in SW620-LCN2, RKO-LCN2 and corresponding control cells, (N, nuclear; C-cytoplasm; P, phosphorylated). (TIF 1603 kb) [file 12943_2016_564_MOESM8_ESM.tif]

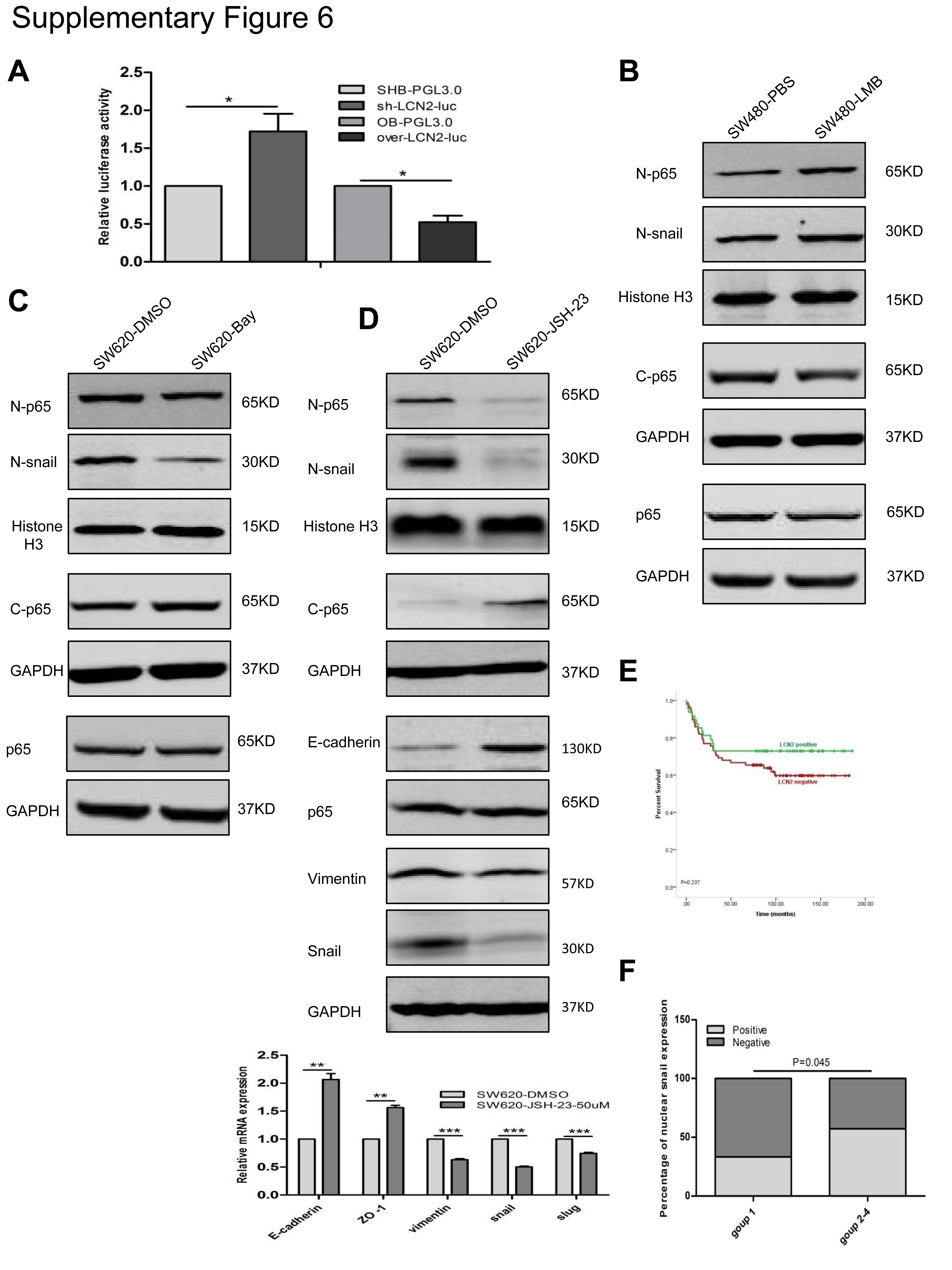

Supplement: Additional file 9: Figure S6. — (A) Quantification of the fold change of luciferase promoter reporter assays of NF-κB in LCN2-knockdown and LCN2-overexpressing cells in 293FT cells. (B,C) p65 and snail (total, nuclear and cytoplasm) expression of SW480 cells treated with LMB (20nM for 10 h) (B) and SW620 cells treated with Bay11-7082 (50 μM for 3 h) (C). (D) Western blots and quantification of EMT markers, p65 (nuclear and cytoplasm) expression in SW620 cells treated with JSH-23 (50 μM for 1 h), (N, nuclear; C-cytoplasm). (E) Survival analysis (Kaplan-Meier method, log-rank test) of LCN2 expression in CRC patients (n = 126, P = .237). (F) Correlation between LCN2/NF-κB expression and nuclear snail expression in clinical samples (group 1: LCN2 (+)/NF-κB (−), total number = 21, 7 cases with positive snail; group 2–4: other groups, total number = 103, 59 cases with positive snail). Values shown are the mean ± SD from at least three independent experiments. * P < .05, ** P < .01, *** P < .001. (TIF 2481 kb) [file 12943_2016_564_MOESM9_ESM.tif]

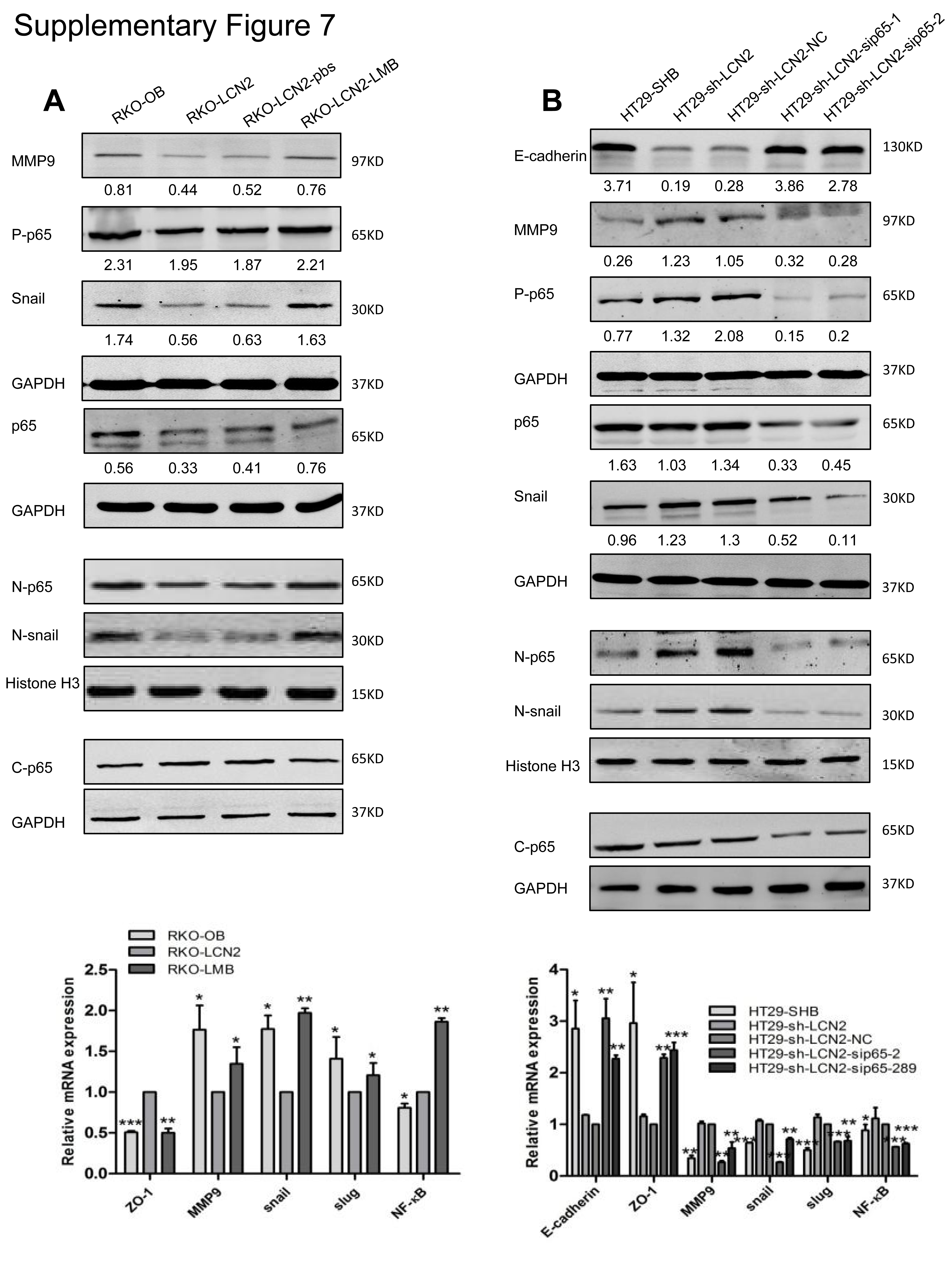

Supplement: Additional file 10: Figure S7. — Association between LCN2 and NF-ĸB/snail pathway in the indicated cells. (A) Changes of MMP9, p65 and snail expression in RKO-LCN2 cells and quantification of relative mRNA expression of EMT key proteins after treatment with LMB (40nM, 6 h) in RKO-LCN2 cells. (B) Changes of EMT key proteins, p65 expression and quantification of relative mRNA expression of EMT key proteins after transfection with specific siRNA (50nM, 48 h) of NF-ĸBp65 in HT-29-sh-LCN2 cells, (N, nuclear; C, cytoplasm; P-phosphorylated). The grey value ratios of the corresponding proteins/GAPDH were shown. Values shown in real-time PCR assay are the mean ± SD from at least three independent experiments. * P < .05, ** P < .01, *** P < .001. (TIF 4190 kb) [file 12943_2016_564_MOESM10_ESM.tif]

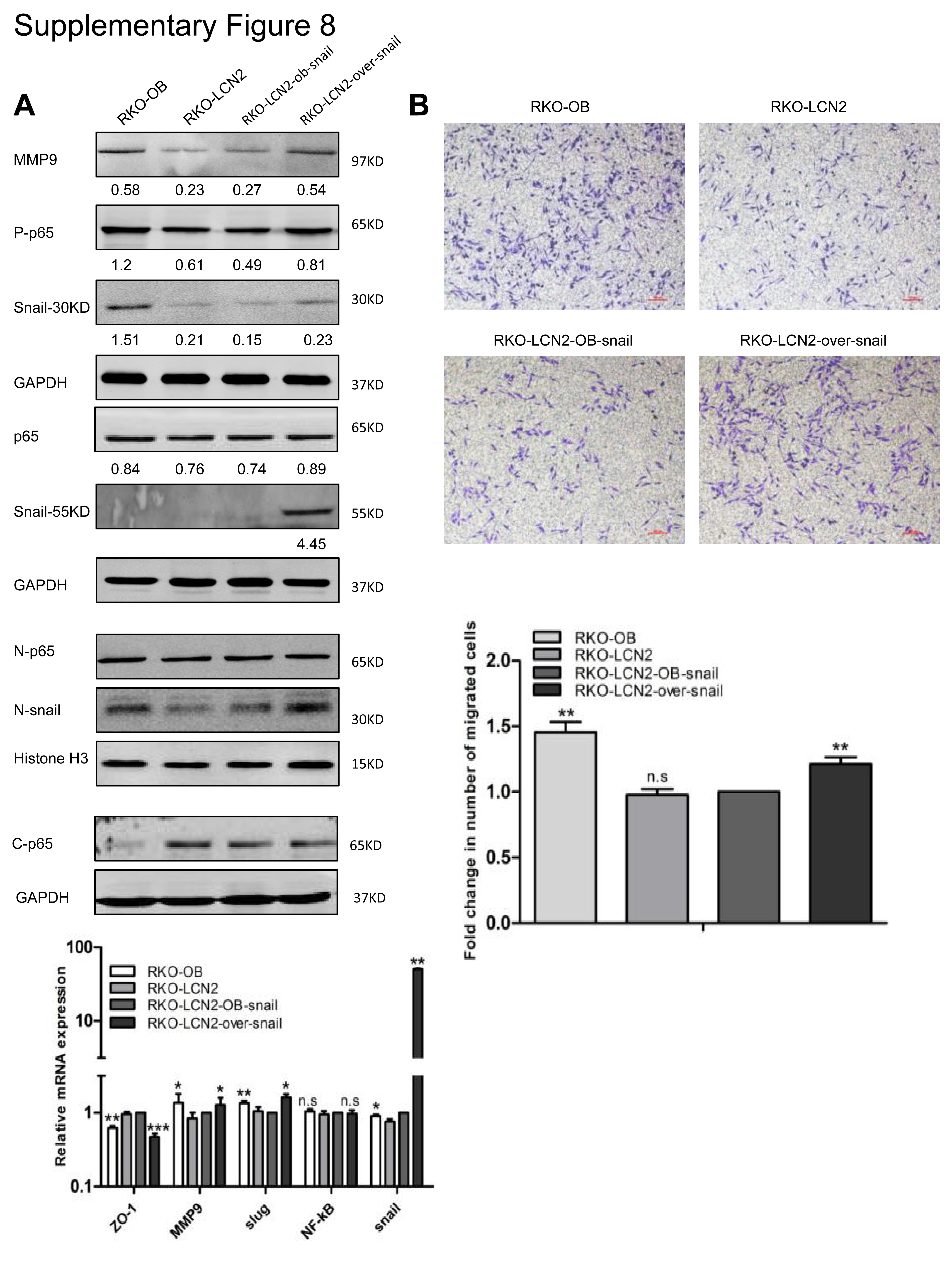

Supplement: Additional file 11: Figure S8. — EMT and migration changes depend on the LCN2/NF-κB/Snail pathway in RKO-LCN2 cell lines. (A) Changes of MMP9, snail and p65 expression assessed by western blots and quantification of relative mRNA expression of EMT key proteins after restoring snail expression in RKO-LCN2 cells. Snail protein (55KD) is a fusion protein with green fluorescence protein (GFP). The grey value ratios of the corresponding proteins/GAPDH were shown. (B) Migration assay and quantification of migration at OD value of 570 nm with a microplate reader after overexpression of snail in RKO-LCN2 cells. Values shown in real-time PCR assay are the mean ± SD from at least three independent experiments., (N, nuclear; C, cytoplasm; P-phosphorylated; over, overexpression). * P < .05, ** P < .01, *** P < .001. Scale bars, 100 μm. (TIF 6141 kb) [file 12943_2016_564_MOESM11_ESM.tif]
